# Enhancing low-light images with MSHCDI-Net: A multi-scale hybrid cross-domain interaction approach

**DOI:** 10.1371/journal.pone.0352326

**Published:** 2026-07-17

**Authors:** Bin Chen, Peitao Li, Chaobing Zheng, Meng Jia, Shiqian Wu

**Affiliations:** 1 School of Electronic Information/Wuhan University of Science and Technology, Wuhan, Hubei, China; 2 College of Mechanical and Electrical Engineering/Xinxiang University, Xinxiang, Henan, China; Dayananda Sagar College of Engineering, INDIA

## Abstract

Low-light image enhancement aims to improve visual visibility and perceptual quality under challenging illumination conditions. However, conventional convolutional neural networks (CNNs) are inherently limited in modeling long-range dependencies due to their restricted receptive fields, which often leads to insufficient global context modeling and suboptimal restoration results. To address this limitation, we propose MSHCDI-Net, a Multi-Scale Hybrid Cross-Domain Interaction Network that effectively integrates CNN and Transformer branches to jointly capture local texture details and global contextual relationships. Specifically, the proposed framework adopts a hierarchical encoder–decoder architecture to perform multi-scale feature extraction and progressive reconstruction. A cross-domain interaction mechanism is introduced to facilitate effective information exchange between convolutional and Transformer representations across multiple resolutions, enabling complementary modeling of fine-grained structures and long-range dependencies. Through adaptive feature fusion and multi-scale guidance, the network achieves improved structural consistency and detail restoration in low-light scenes. Extensive experiments on several public benchmarks demonstrate the effectiveness of the proposed method. MSHCDI-Net achieves 23.45 dB PSNR / 0.848 SSIM on LOL-v1, 23.74 dB / 0.910 SSIM on LOL-v2-synthetic, and 22.24 dB / 0.868 SSIM on LOL-v2-real, demonstrating competitive performance in both quantitative metrics and visual quality.

## 1. Introduction

Low-light image enhancement (LLIE) aims to reconstruct bright, clear, and visually natural images from underexposed, noisy, and degraded inputs.This task plays a critical role in many high-level computer vision applications, such as night-time surveillance, autonomous driving, and object detection [1,2]. However, enhancing low-light images remains challenging due to the inherent low signal-to-noise ratio of dark regions, spatially uneven illumination distribution, and tendency toward local overexposure during enhancement, which easily induce severe noise amplification, loss of fine details, and distortion.

Traditional LLIE approaches mainly include grayscale stretching techniques and Retinex-based models. Grayscale stretching methods, such as histogram equalization [3] and gamma correction, enhance contrast by adjusting the global or local pixel intensity distribution. Although computationally efficient, these approaches often introduce over-enhancement and color distortion. In contrast, Retinex-based methods, inspired by the Land–McCann Retinex theory [4], aim to decompose images into illumination and reflectance components. While such methods can better adjust dynamic range, accurately estimating illumination remains difficult, often leading to halo artifacts and inconsistent visual perception. Recent frequency-domain methods [5,6] attempt to alleviate these limitations by decomposing images into structural and texture components, thereby improving noise suppression performance and fine detail preservation.

With the rapid advancement of deep learning, data-driven LLIE methods have achieved significant progress. Convolutional neural networks (CNNs) [7] have demonstrated strong capability in capturing local textures and edges, enabling effective restoration of spatial details. However, due to their inherently limited receptive fields, CNN-based models struggle to capture long-range dependencies, which are crucial for modeling global illumination consistency. To address this limitation, several studies have incorporated physical priors into deep networks. For example, Li et al. [8] introduced a Retinex-inspired framework that decomposes images into illumination and reflectance, improving illumination modeling. Nevertheless, modeling large-scale illumination variations in complex real-world low-light scenes remains a formidable challenge for purely convolutional architectures.

Recently, Transformer-based architectures [9] have been widely applied into low-level vision tasks due to their ability to model long-range dependencies through self-attention mechanisms. Methods such as Uformer [10], Restormer [11], and LLFormer [12] demonstrate improved global context modeling and enhanced feature propagation. However, vanilla Transformer architectures often suffer from high computational cost and limited ability to capture fine local textures, which are essential for low-light image enhancement.

To fully exploit the complementary advantages of CNNs and Transformers, hybrid architectures have recently emerged. For example,HVI-CIDNet [13] integrates CNN and Transformer structures for more accurate illumination modeling. Nevertheless, existing hybrid approaches often lack sufficiently deep cross-domain feature interaction, which limits the effective integration of global context and local structural details.

**Fig 1:**
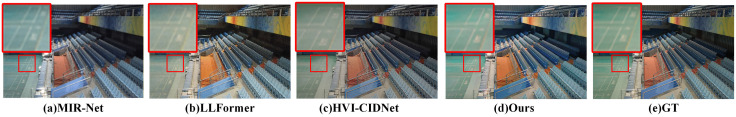
Comparison of results from four low-light image enhancement algorithms. From **(a)** to **(d)**, the images correspond to the results produced by MIR-Net [14], LLFormer [12], HVI-CIDNet [13] and ours, respectively. The **(e)** represents the ground truth. It can be observed that the proposed algorithm is capable of preserving detail information.

As visualized in Fig 1, existing methods still exhibit noticeable limitations. CNN-based methods may suffer from color shifts and detail loss, while Transformer-based approaches may blur fine textures despite superior global illumination consistency. Even hybrid models sometimes fail to fully exploit the complementary strengths of both architectures. Therefore, how to effectively integrate CNN-based local feature learning with Transformer-based global context modeling while achieve efficient cross-domain feature interaction remains a challenging open problem in the field of LLIE.

**Fig 2:**
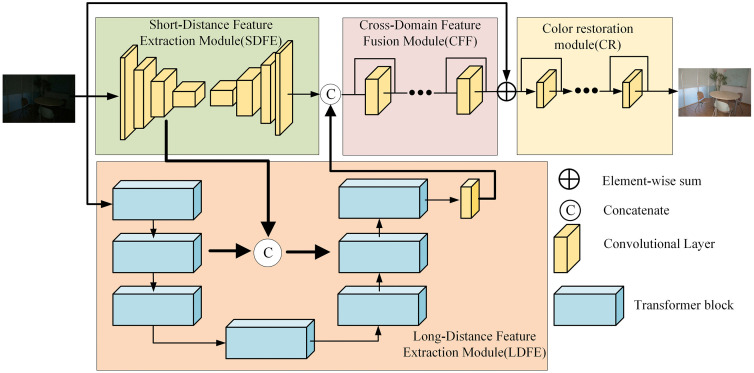
The MSHCDI-Net comprises four modules:the Short-Distance Feature Extraction Module (for local feature extraction), the Long-Distance Feature Extraction Module (for global feature extraction), the Cross-Domain Feature Fusion Module (for efficient feature fusion), and the Color Restoration Module (for high-quality color recovery).

To tackle the aforementioned critical challenges—insufficient balance between local detail preservation and global illumination consistency, shallow cross-domain feature interaction in existing hybrid architectures, and prevalent color distortion in low-light enhancement—we propose a novel Multi-Scale Hybrid Cross-Domain Interaction Network (MSHCDI-Net) for high-quality low-light image enhancement. As illustrated in Fig 2, the proposed dual-branch framework deeply integrates CNN-based local feature extraction and Transformer-based global context modeling (unlike conventional shallow fusion), with four innovative components targeting specific limitations of existing methods: (1) a Short-Distance Feature Extraction (SDFE) module to refine local textures and alleviate Transformer-induced blurring; (2) a Long-Distance Feature Extraction (LDFE) module to model global illumination and overcome CNN’s limited receptive field; (3) a novel Cross-Domain Feature Fusion (CFF) module to realize deep hierarchical interaction between local and global features; (4) a dedicated Color Restoration (CR) module to mitigate color distortion, an underaddressed issue in LLIE.

The main contributions of this work are summarized as follows:

- A novel hybrid framework, MSHCDI-Net, is presented for low-light image enhancement, aiming to address the limited interaction between CNN-based local representations and Transformer-derived global contexts in existing hybrid architectures. A Cross-Domain Feature Fusion (CFF) module is introduced to enable deep, hierarchical, and adaptive cross-domain feature interaction, facilitating effective information exchange and strengthening complementary representation learning.
- A lightweight Color Restoration (CR) module is further developed to alleviate color distortion commonly observed in low-light enhancement. The module enhances chromatic consistency and color fidelity while introducing negligible computational overhead, thereby improving visual realism and complementing the limitations of current methods.

The remainder of this paper is organized as follows. Section 2 reviews related work on low-light image enhancement. Section 3 presents the proposed method. Section 4 reports experimental results and analysis. Finally, Section 5 concludes the paper.

## 2. Related works

Low-light image enhancement has long been vital for applications in photography, surveillance, and autonomous systems. Existing methods can be broadly categorized into model-based and data-driven approaches, each with its own strengths and limitations.

**Model-based low-light image enhancement** methods, such as histogram equalization and gamma correction, enhance dynamic range and contrast by adjusting pixel intensity distribution. However, these methods often introduce visual artifacts or color distortion due to their global and non-adaptive adjustment strategies. Retinex-based methods [15,16] decompose images into illumination and reflectance, providing more natural enhancement. However, their performance is limited by simplified illumination assumptions, which reduces their robustness in complex real-world low-light scenes with spatially non-uniform lighting.

**Data-driven Low-light Image Enhancement** methods have leveraged deep learning for more adaptive and perceptually consistent enhancement. Retinex-Net [17], KinD [18], and EnlightenGAN [19] demonstrate the power of CNN-based architectures in capturing structural detail and suppressing noise. Nevertheless, the inherent local receptive fields of CNNs restrict their ability to model global illumination consistency, especially under spatially varying illumination, often resulting in uneven enhancement.

To address these issues, Vision Transformers (ViTs) [20] have been introduced for image restoration. Models such as MAE [21], Uformer [10], and Restormer [11] leverage global self-attention to enhance feature propagation and global contextual understanding. However, vanilla Transformer designs often incur high computational costs and may overlook fine textures.

Recent efforts have explored hybrid CNN–Transformer architectures, such as LightingFormer [22], RSTB-DRB [23], and SNR-Net [24]. Although these models introduce cross-domain feature fusion, they often lack deep multi-scale interaction, leaving the full potential of hybrid designs underexplored.

Despite progress, current methods still face challenges in balancing noise suppression, fine-grained local detail preservation, and global illumination consistency in complex real-world low-light scenes. To address these intertwined challenges, our proposed MSHCDI-Net fully integrating CNNs and Transformers through a multi-scale hybrid structure that enhances both local detail and global consistency.

## 3. The proposed method

To enhance both local texture detail and overall image consistency, this paper proposes MSHCDI-Net, an end-to-end low-light image enhancement (LLIE) model based on a hybrid architecture that integrates Convolutional Neural Networks (CNNs) and Vision Transformers. CNNs automatically learn spatial hierarchical features through multiple convolutional layers, enabling the effective extraction of local image details. In contrast, Transformers utilize self-attention mechanisms to model global dependencies, making them well-suited for capturing long-range contextual information. By leveraging the strengths of both CNNs and Transformers, MSHCDI-Net achieves a favourable balance between computational efficiency and enhancement performance across LLIE tasks. Moreover, this hybrid design facilitates an effective trade-off between latency and accuracy.

The overall architecture of MSHCDI-Net, depicted in Fig 2, comprises four key modules: (1) a Short-Distance Feature Extraction Module, which leverages CNNs to effectively capture local image features; (2) a Long-Distance Feature Extraction Module, which employs a multi-head self-attention mechanism to model global dependencies across the image; (3) a Cross-Domain Feature Fusion Module, which is proposed to efficiently fuse the heterogeneous features and fully exploit the complementary advantages of CNN and Transformer branches; and (4) a Color Restoration Module, responsible for reconstructing high-quality, visually realistic outputs. Detailed descriptions of each module are provided in the following sections.

### 3.1 Short-Distance Feature Extraction Module (SDFE)

**Fig 3:**
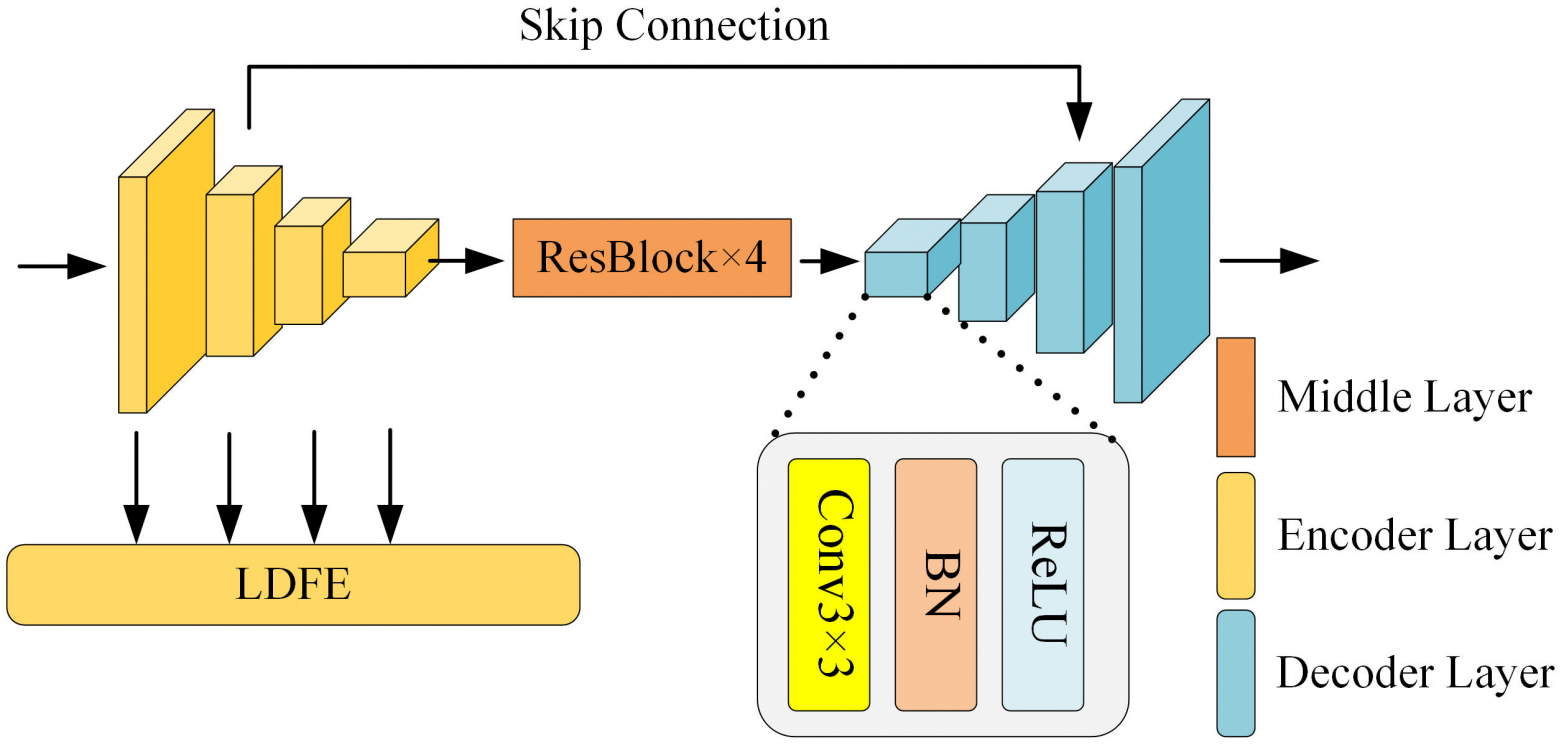
Short-Distance Feature Extraction Module(SDFE), The SDFE module consists of convolutional layers, adopting skip connections between encoder and decoder and embedding residual blocks in intermediate layers.

The Short-Distance Feature Extraction Module (SDFE) adopts an optimized UNet [25] structure, as shown in Fig 3. The optimized UNet retains skip connections and the encoder-decoder structure but replaces intermediate linear layers with residual blocks. Each layer in the encoder and decoder consists of a 3×3 convolution, Batch Normalization (BN), and ReLU activation. The 3×3 convolution helps capture local features while reducing parameters, contributing to a lightweight network. BN stabilizes the input distribution, accelerates training, and improves generalization. ReLU introduces non-linearity to enable complex mappings and mitigating gradient vanishing.

Skip connections transfer features from the encoder to the decoder, preserving spatial information for structural consistency and detail fidelity. The decoder fuses high-level semantic features with low-level details via skip connections, achieving accurate and visually coherent reconstructions. Residual blocks enhance deep feature learning, crucial for recovering subtle visual cues in low-light images. The SDFE module’s operation is defined in Equation 1:


$$\begin{array}{c} F_{SDFE}=D_{SDFE}(E_{SDFE}(I_{low})) \end{array}$$ (1)

where *D* and *E* represent the decoder and encoder of the UNet, respectively, $F_{SDFE}$ is the output of the module, and $I_{low}$ is the input low-light image.

### 3.2 Long-Distance Feature Extraction Module (LDFE)

**Fig 4:**
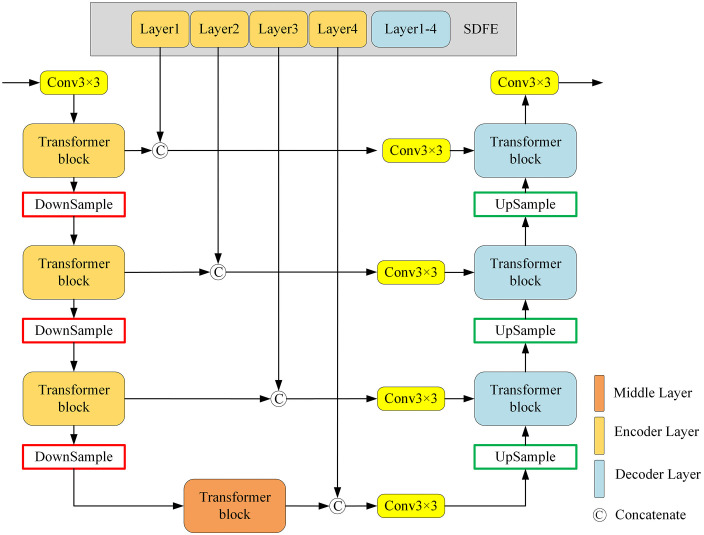
Long-Distance Feature Extraction Module(LDFE), which integrates Transformers into the U-shaped architecture, allowing each level of the Transformer encoder to share features with the encoder from the SDFE module.

As shown in Fig 4, the Long-Distance Feature Extraction Module adopts a U-shaped structure with 10 Transformer blocks to help efficiently capture and process global features. The module consists of a hierarchical encoder and decoder. Initially, the input image is embedded via a convolutional layer, where overlapping patches are generated through a convolutional operation (kernel stride = 1, padding applied). This approach preserves inter-patch continuity, minimizing the loss of local contextual information at boundaries, which is beneficial for maintaining texture and edge continuity in image enhancement tasks.

These patch representations are fed into Transformer blocks based on Vision Transformer (ViT) [20], where we adopt the Transposed self-attention mechanism from Restormer [11].

Compared with vanilla self-attention, windowed self-attention, and non-local attention, Transposed self-attention has three specific advantages for low-light image enhancement:

1. **Computational efficiency**: Vanilla self-attention suffers from *O*(*N*^2*C*^) complexity, which is too heavy for high-resolution low-light images. Transposed self-attention reshapes queries and keys to reduce the complexity to *O*(*NC*^2^), enabling efficient global modeling.
2. **Local-global feature synergy**: Window-based attention breaks long-range dependencies and harms illumination consistency. Transposed self-attention retains full global attention while combining local details via convolutions in *QKV* projection, avoiding over-smoothing.
3. **Attention stability**: Non-local attention easily amplifies noise in dark regions. Transposed self-attention uses a scaling factor α before Softmax to stabilize attention scores and reduce noise-induced artifacts.

Given the input feature map $Y\in {\mathbb{R}}^{H\times W\times C}$, we initially generate the query *Q*, key *K*, and value *V*, which integrate local context information.Specifically, we first use the pointwise convolution 1×1 of $W_{(\cdot )p}$ to aggregate the pixel – level cross – channel context, followed by depthwise convolution 3×3 of $W_{(\cdot )d}$ to encode the channel – level spatial context, denoted as $Q=W_{Qd}\cdot W_{Qp}\cdot Y$, $K=W_{Kd}\cdot W_{Kp}\cdot Y$, $V=W_{Vd}\cdot W_{Vp}\cdot Y$. To reduce the computational complexity of the original self – attention, we reshape the projections of the query, key and value to dimensions $\hat{𝐐}\in {\mathbb{R}}^{HW\times C}$, $\hat{𝐊}\in {\mathbb{R}}^{C\times HW}$, $\hat{𝐕}\in {\mathbb{R}}^{HW\times C}$. The attention map, with a size of ${\mathbb{R}}^{\hat{C}\times \hat{C}}$, is generated via dot-product interaction between $\hat{𝐐}$ and $\hat{𝐊}$. The calculation process of the attention scores follows the formula below:


$$\begin{array}{c} \text{Attention}(\hat{𝐐},\hat{𝐊},\hat{𝐕})=\hat{𝐕}\cdot \text{Softmax}(\hat{𝐊}\cdot \hat{𝐐}/\alpha ) \end{array}$$ (2)

Where α controls the magnitude of the dot – product between $\hat{𝐐}$ and $\hat{𝐊}$ and then applies the Softmax function.After obtaining the attention output, we apply a 1×1 convolution operation and add the result to the input feature map *X* to obtain the final output $\hat{X}$.


$$\begin{array}{c} \hat{X}=W_{p}\text{Attention}(\hat{𝐐},\hat{𝐊},\hat{𝐕})+X \end{array}$$ (3)

Recent research, such as Restormer [11], has shown that enhancing the Transformer’s feed-forward network with a gating mechanism improves performance in image reconstruction tasks. Consequently, each Transformer block integrates a Multi-Layer Perceptron (MLP) with a gating mechanism for feature transformation and nonlinear mapping. Layer Normalization is used to help ensure training stability and accelerate convergence.

### 3.3 Multi-Scale Cross-Domain Feature Fusion via the LDFE Modules

To help effectively leverage structural and semantic information across multiple spatial scales, we employ a multi-scale feature extraction, fusion, and reconstruction strategy based on two heterogeneous architectures.

**Multi-Scale Feature Extraction:** The LDFE encoder is composed of hierarchical Transformer blocks. At each level *i*, features are transformed and then downsampled to reduce spatial resolution while increasing channel dimensionality, enhancing semantic abstraction. In parallel, the SDFE encoder extracts localized features $F_{\text{en}SDFE}^{i}$ at the same scale via convolutional layers.

**Fig 5:**
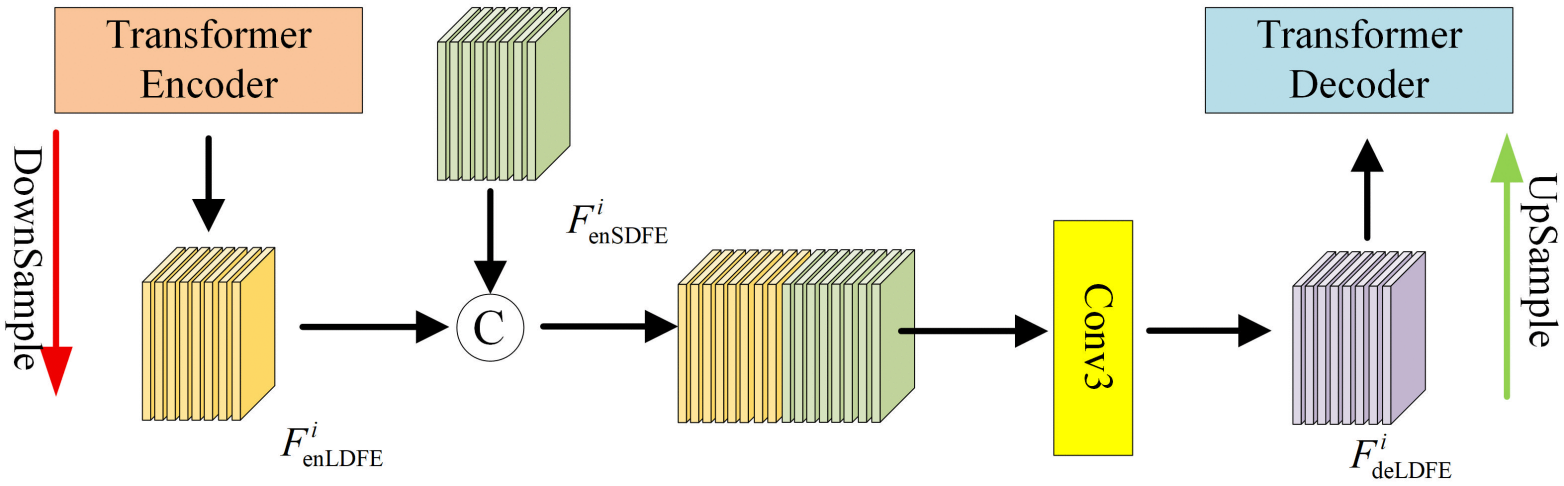
Illustration of the encoding and decoding processes at different levels in the LDFE module. The encoder-decoder structure hierarchically processes multi-resolution feature maps and fuses global and local features from both the LDFE and SDFE modules.

**Cross-Scale Feature Fusion:** As shown in Fig 5, a hierarchical fusion mechanism is employed to integrate global features $F_{\text{en}LDFE}^{i}$ and local features $F_{\text{en}SDFE}^{i}$. These are concatenated along the channel dimension and fused via a 3×3 convolution:


$$\begin{array}{c} F_{LDFE}^{j}=D_{LDFE}\{Conv3[(E_{LDFE}^{i}(I_{in}))concat(E_{SDFE}^{i}(I_{\text{in}}))]\} \end{array}$$ (4)

where $F_{\text{LDFE}}^{j}$ denotes the decoder output at level *j*, and *I*_in_ is the input feature map at level *i*.

**Multi-Scale Reconstruction:** The decoder adopts a mirror structure corresponding to the encoder. Upsampling modules progressively restore spatial resolution, and fused encoder features are integrated at each level. This design preserves both global structure and local detail. A final convolution projects the reconstructed multi-scale features back to the image domain.

Overall, this strategy enables adaptive balance between structural consistency and texture fidelity, making it highly effective for low-light image enhancement in complex visual environments.

### 3.4 Cross-Domain Feature Fusion Module(CFF)

**Fig 6:**
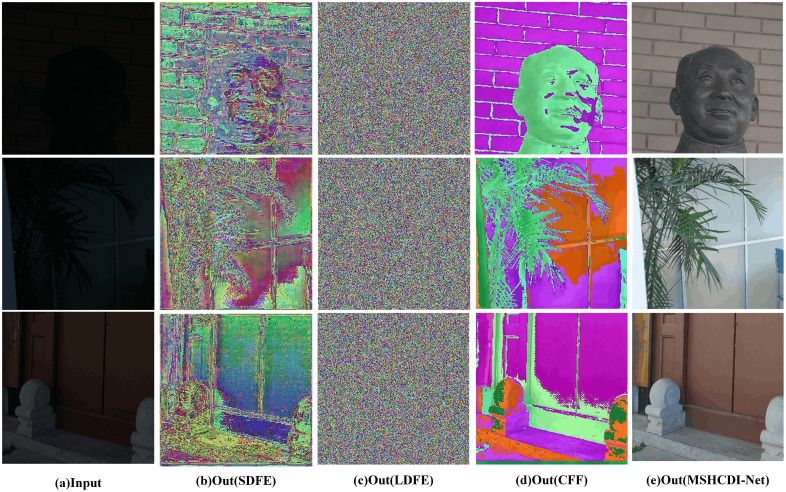
To gain a more intuitive understanding of the semantic differences between the outputs of the SDFE and LDFE modules, and to assess the fusion effect achieved by the CFF module, we extracted the output features from these modules within the MSHCDI-Net architecture.

As shown in Fig 6, the SDFE module (CNN-based) captures local image details through convolutional kernels, with shallow layers primarily capture low-level features (edges, textures) and deeper layers infer semantic structures. This preserves spatial correspondence and image details. In contrast, the LDFE module, based on the Transformer architecture, models long-range dependencies through self-attention mechanisms. Although it excels at capturing holistic contextual structures, the generated features tend to be more abstract and may lack explicit spatial correspondences.

Experimental results indicate noticeable semantic and spatial mismatches between the modules’ outputs. Basic fusion approaches (e.g., concatenation or element-wise addition) often fail to effectively combine their complementary features and may cause misalignment.

**Fig 7:**
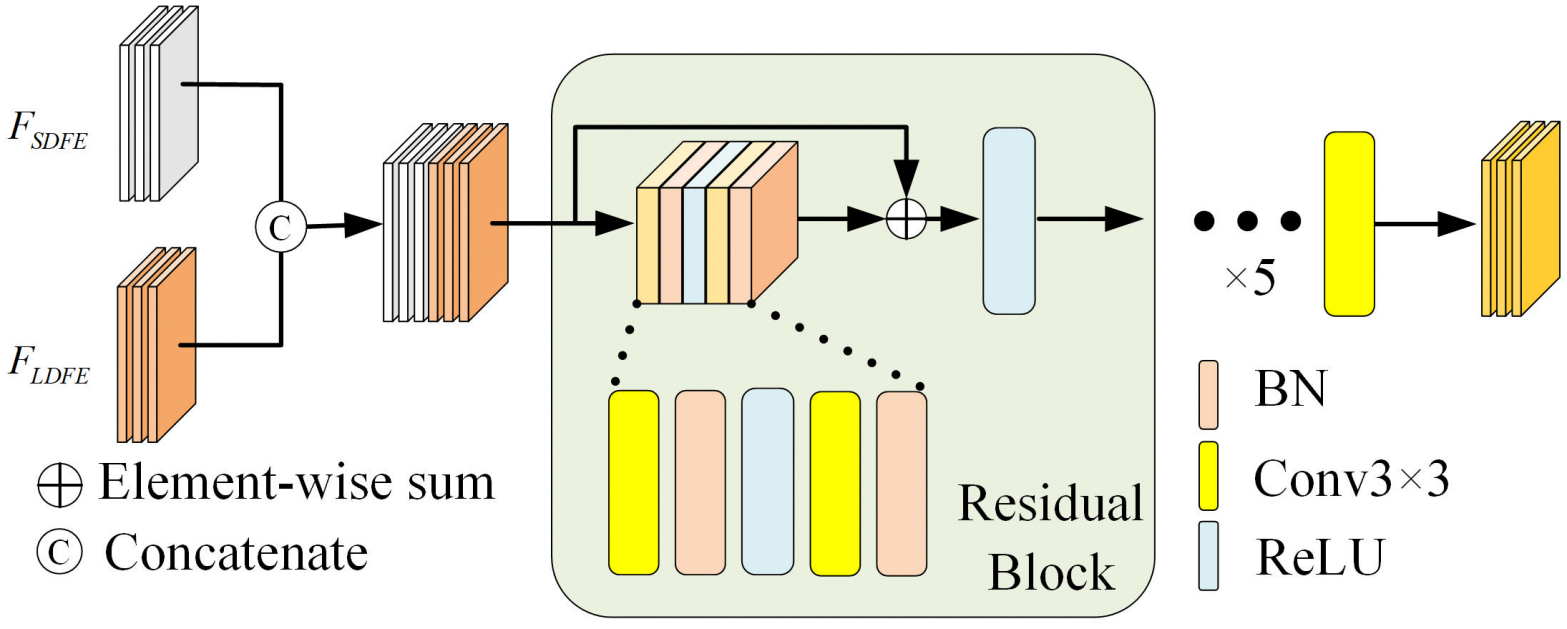
The Cross-Domain Feature Fusion Module (CFF) concatenates the feature maps from the LDFE and SDFE modules, followed by five sequential residual blocks comprising 3 × 3 convolutions, which are designed for semantic alignment and feature fusion.

To mitigate this issue, we designed an iterative residual network employing 3×3 convolutions, as depicted in Fig 7.The specific process is presented in the following formula. Here, δ represents the ReLU activation function, BN represents batch normalization, and $x_{in}$ represents the input of the residual module.


$$\begin{array}{c} \text{Residual}{\;}_{CFF}=\delta \{\text{BN}[Conv3(\delta (\text{BN}(Conv3(x_{in}))))]+x_{in}\} \end{array}$$ (5)

This network is designed to integrate the global and local semantics of the input image. Through iterative processing by multiple residual blocks, the network gradually alleviates the semantic discrepancies between the outputs of the SDFE and LDFE modules. Each layer’s convolution operations help adjust and align the feature space, thereby facilitating the effective fusion of the two types of features at a higher level.The specific operation process of the CFF module is presented in the following formula. In this formula, *n* denotes the number of iterations of the residual block.


$$\begin{array}{c} F_{CFF}=Conv3\{\text{Residual}{\;}_{CFF}[(F_{LDFE})\text{Concat}(F_{SDFE})]\times n\},n=5 \end{array}$$ (6)

**Fig 8:**
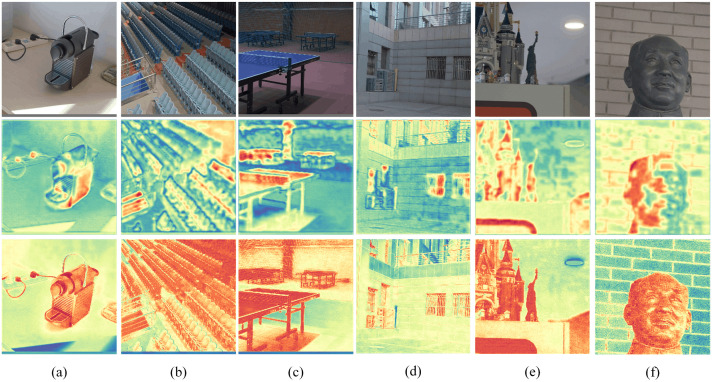
Illustrates the visualization of attention maps after being processed by the Cross-Domain Feature Fusion (CFF) module. Our experimental results unequivocally demonstrate the effectiveness of the CFF module in handling diverse scenes and objects. As shown in the figure, it compares the ground truth (GT), attention maps before processing through the CFF module, and attention maps after processing. This comparison highlights the enhancement achieved by the module in accurately capturing relevant features across different domains.

By extracting and visualizing the attention distribution maps generated by the CFF module and overlaying them onto output feature maps (Fig 8a–8f), we observe that post-fusion feature maps show clearer and more accurate detail restoration compared to pre-fusion states. Prior to fusion, the model’s attention mechanism was confined to local regions for targets such as coffee makers (Fig 8a), chairs (Fig 8b), ping-pong tables (Fig 8c), windows (Fig 8d), castles (Fig 8e), and sculpture heads (Fig 8f), failing to capture the complete object structure. This localized attention limits the holistic feature extraction and global-information utilization for detail enhancement.

The CFF module improves attention mechanisms by expanding focus from local to global scales. Post-fusion attention maps exhibit stronger capability to identify and process semantically correlated image regions. For example, attention in Fig 8a extended to the full coffee maker and contextual surroundings, enabling accurate context capture; Fig 8f showed holistic focus on sculpture heads, improving detail fidelity; and Fig 8c highlighted integrated attention to ping-pong table components (net, boundary lines, legs), enhancing recognition robustness.

In complex scenes, the CFF module also promotes better spatial layout understanding. For instance, in Fig 8b (multi-chair scenes), pre-fusion attention was fragmented across individual chairs, whereas post-fusion attention aligned to row contours, clarifying structural organization. In Fig 8e, castle representations benefited from attention spanning local architectural details (towers, arches) and global structural contexts, enabling effective extraction of complex feature hierarchies.

In summary, the CFF module enhances both local detail recognition and global structure understanding via semantic information fusion, enabling the model to achieve improved accuracy and robustness in complex low-light image enhancement tasks.

### 3.5 Color Restoration Module(CR)

**Fig 9:**
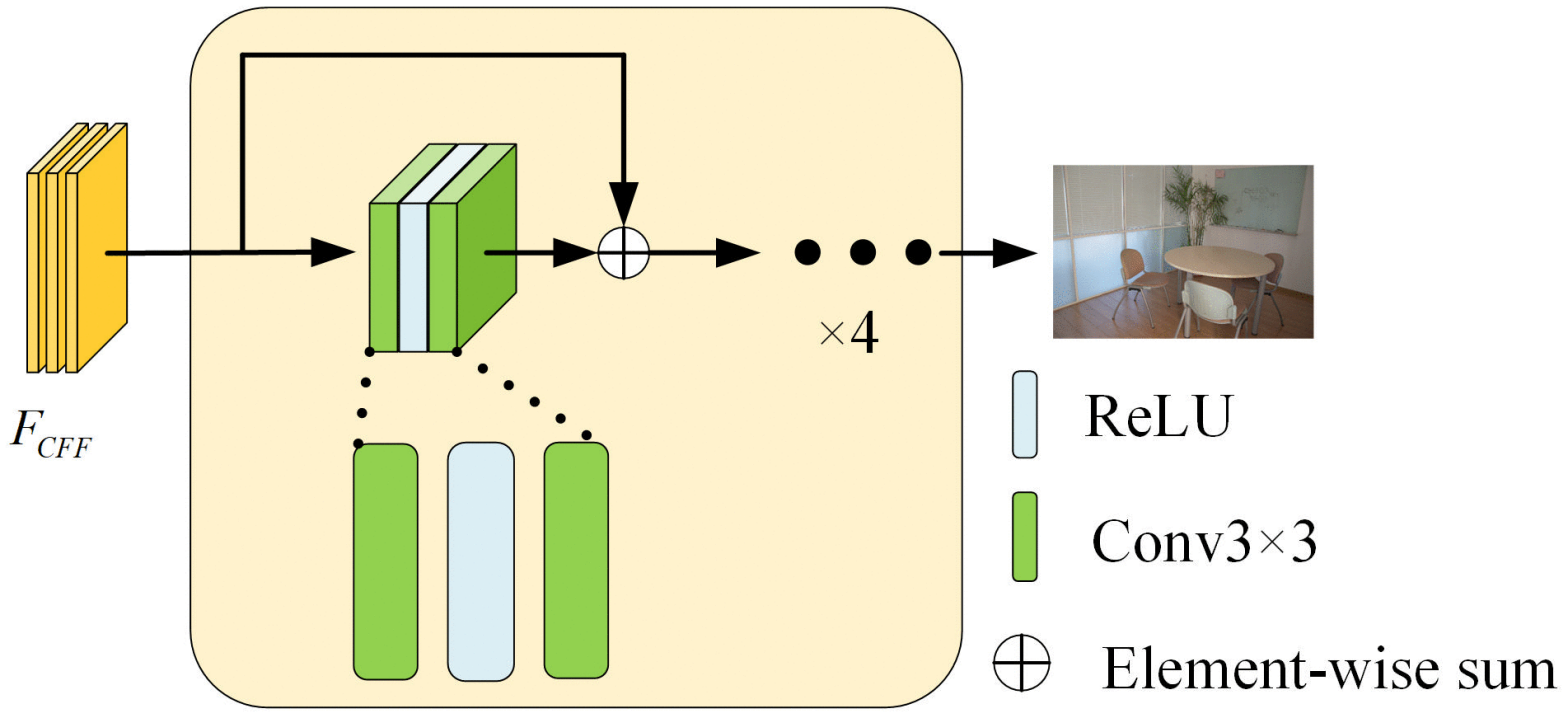
The Color Restoration Module (CR) employs four consecutive 1 × 1 convolutional residual blocks to perform color restoration on the feature maps fused by the CFF module.

We introduced a residual structure that is based on the stacking of 4 convolutional layers. As illustrated in Equation (7) and Fig 9, this structure aims to optimize and enhance the color representation of the image.


$$\begin{array}{c} \text{Residual}{\;}_{CR}=\{Conv1[\delta (Conv1(x_{in}))]\}+x_{in} \end{array}$$ (7)

The output of the CR module, which serves as the final output of the MSHCDI-Net model, is presented in Equation 8. In this equation, *n* = 4 indicates that the iteration is carried out 4 times.


$$\begin{array}{c} F_{CR}={\text{Residual}}_{CR}(F_{CFF})\times n,n=4 \end{array}$$ (8)

**Fig 10:**
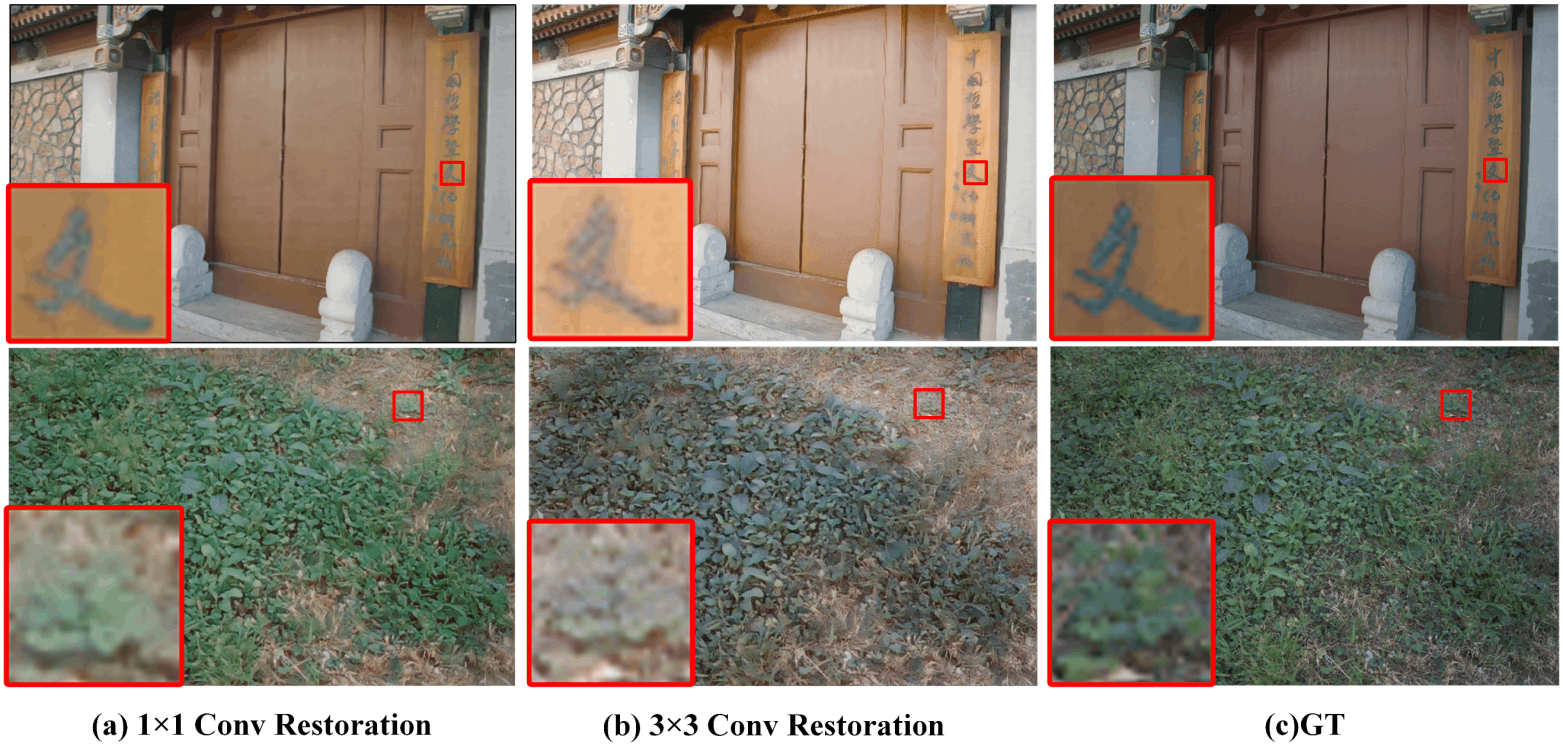
The choice of different convolutional kernel sizes on color recovery. Results indicate that the use of 1×1 kernel yields color recovery that is significantly closer to the ground truth. In contrast, employing kernel size 3×3 results in noticeable color distortion, accompanied by a loss of fine details.

The 1×1 convolution maintains spatial structure through per-pixel operations without spatial aggregation, making it particularly effective for color recovery by preserving inter-pixel color differences. It further enhances color representation in multi-channel spaces like RGB through adaptive channel weighting via linear cross-channel fusion. In contrast, larger kernels (e.g., 3×3) induce spatial smoothing that, while useful for initial feature extraction, leads to color blurring in high-contrast regions during final recovery stages. Both theoretical analysis and experimental results demonstrate the superior performance of 1×1 convolutions in color restoration tasks, achieving more precise color recovery and higher-quality image reconstruction. Fig 10 illustrates that employing larger convolution kernels for color recovery induces excessive spatial smoothing, as evidenced by text blurring in the first row and color distortion within the red-boxed regions (second row).

### 3.6 Loss function

Since both the *L*_2_ and *L*_1_ loss functions are point-wise metrics, they tend to produce overly smooth outputs, which may lead to the loss of fine details. To mitigate this issue, the Charbonnier loss [26] and Perceptual loss [27] are incorporated during the training process. The Charbonnier loss, being a differentiable variant of the *L*_1_ norm, exhibits strong robustness to outliers (such as noise) and unlike the *L*_2_ loss, does not overly penalize large deviations. This property enables the preservation of more intricate image details. In addition, the Perceptual loss emphasizes texture representation, thereby facilitating the generation of sharper and more visually realistic outputs.


$$\begin{array}{c} L_{c}=\frac{\sum_{\left(i,j\right)\in valid}\sqrt{{\left(I^{\prime }\left(i,j\right)-\hat{I}\left(i,j\right)\right)}^{2}+\varepsilon^{2}}}{N} \end{array}$$ (9)

Here, $I^{\prime }$ denotes the predicted image and $\hat{I}$ denotes the ground-truth image. Charbonnier Loss introduces a small positive number ε to avoid numerical instability issues. Since the dataset has been padded, the loss is calculated only for the valid region and is divided by *N* (the total number of pixels in the valid region) to eliminate imbalances caused by varying sizes of the effective region. This ensures stable gradient calculation during backpropagation, thereby making the training process more balanced and effective.


$$\begin{array}{c} L_{p}=\frac{1}{N}\sum_{(i.j)\in valid}{\left\|\phi_{n}(I^{\prime }(i,j))-\phi_{n}(\hat{I}(i,j))\right\|}_{2} \end{array}$$ (10)

*n* represents the *n* -th layer’s features in the pre-trained VGG model, and the final complete loss function can be expressed as:


$$\begin{array}{c} L=L_{c}+0.1\times L_{p} \end{array}$$ (11)

By combining Charbonnier Loss and Perceptual Loss, the proposed loss function helps enhances the preservation of fine details and improve visual fidelity, while concurrently maintaining training stability.

## 4. Experiment results

### 4.1 Datasets and implementation details

We evaluated the proposed method on three commonly used low-light image enhancement benchmarks: LOL-v1, LOL-v2-real, and LOL-v2-synthetic. LOL-v1 contains paired low-light and normal-light images and is widely used for supervised LLIE evaluation. LOL-v2 includes both real and synthetic subsets, where LOL-v2-real reflects real-world low-light degradation, while LOL-v2-synthetic is generated under controlled illumination conditions. These datasets provide different low-light scenarios for evaluating the enhancement performance, robustness, and generalization ability of the proposed method.

In this section, we first describe the experimental settings, including the baseline methods and evaluation metrics. Subsequently, ablation studies are conducted to demonstrate the effectiveness of the proposed model. Finally, extended experiments are carried out for comprehensive evaluation. Readers are invited to view the electronic version of the full-size figures and zoom in on them to better appreciate the differences among images.

### 4.2 Experimental settings

Our implementation is built upon the PyTorch framework and executed on four NVIDIA GTX 4070 Ti GPUs. To enhance the diversity of the training data, we apply data augmentation techniques, including mirroring and randomly cropping 256*256 patches from each input image. The proposed MSHCDI-Net is trained using the designed loss functions and optimized with the AdamW optimizer employing a batch size of 4. The initial learning rate is set to 10^−4^ and progressively reduced following a cosine annealing schedule.

In this section, the proposed algorithm is compared with KinD[18], EnlightenGAN [19], Retinex-Net [17], MIR-Net [14], SNR-Net [24], LLFormer [12], LEDNet [28] and HVI-CIDNet [13]. These methods cover the main LLIE paradigms. Specifically, Retinex-Net, KinD, MIRNet, and SNR-Net are CNN-based approaches; EnlightenGAN is a GAN-based method; LLFormer is a Transformer-based model; and SNR-Net and HVI-CIDNet further represent hybrid architectures combining CNN and Transformer components. This selection enables a comprehensive evaluation across different design philosophies, facilitating a balanced and fair comparison with state-of-the-art LLIE methods.

### 4.3 Comparisons with different algorithms

**Fig 11:**
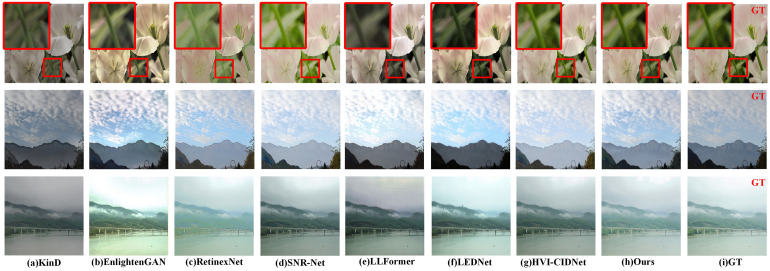
Comparative analysis of low-light image enhancement methods on LOL-v2-synthetic. From left to right, the images represent the results of KinD, EnlightenGAN, Retinex-Net, SNR-Net, LLFormer, LEDNet, HVI-CIDNet and our proposed method, respectively. The last column shows the ground truth.

Fig 11 presents a comparative analysis on the LOL-v2-synthetic dataset. As shown in the first row, KinD, EnlightenGAN, LLFormer, and LEDNet exhibit insufficient enhancement in low-light regions, with details often appearing blurred. Retinex-Net and SNR-Net, on the other hand, produce results with noticeable color oversaturation. Both the proposed method and HVI-CIDNet achieve superior visual quality; it is noteworthy that both approaches are hybrid models.In the second row, the results indicate that KinD suffers from under-enhancement, while SNR-Net, Retinex-Net, and HVI-CIDNet demonstrate inadequate denoising capabilities, leading to noticeably blurred outputs. In contrast, the proposed method delivers high-quality enhancement across regions of varying brightness. The third row further demonstrates that the proposed method excels in overall brightness restoration, producing results that are more visually consistent with the ground truth (GT) images. In comparison, KinD, SNR-Net, LLFormer, and HVI-CIDNet exhibit obvious deficiencies in recovering appropriate brightness levels

**Fig 12:**
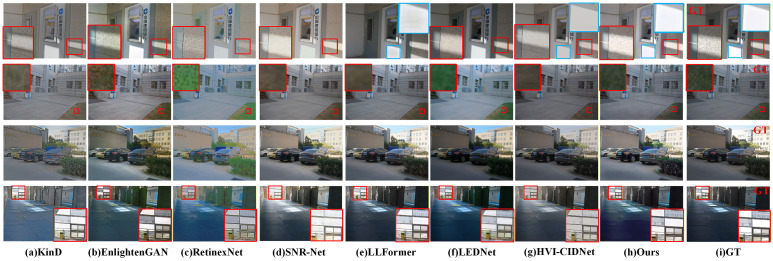
Comparative analysis of low-light image enhancement methods using the LOL-v2-real dataset. From left to right, the images represent the results of KinD, EnlightenGAN, Retinex-Net, SNR-Net, LLFormer, LEDNet, HVI-CIDNet and our proposed method, respectively. The last column shows the ground truth.

Fig 12 presents a comprehensive comparison of our method against several state-of-the-art approaches on the LOL-v2-real dataset. In the first row, as highlighted by red bounding boxes, both KinD and EnlightenGAN produce noticeable artifacts in these challenging areas. Retinex-Net exhibits relatively weak performance in high-contrast regions, resulting in an obvious loss of detail. SNR-Net, LEDNet, and HVI-CIDNet exhibit minor detail loss, while HVI-CIDNet, in particular, shows insufficient brightness restoration in overexposed regions marked with blue boxes. Although LLFormer achieves reasonable brightness recovery, it introduces visible visual artifacts. In contrast, our method delivers more faithful reconstructions. The second row illustrates overall brightness and color restoration performance. Visually, our method aligns most closely with the GT images. In the context of local color restoration (highlighted in red boxes), KinD, EnlightenGAN, SNR-Net, and HVI-CIDNet exhibit inadequate color recovery, while Retinex-Net and LEDNet tend to over-enhance local color information. In the third row, we evaluate performance in scenes with pronounced brightness transitions under real-world conditions. KinD, Retinex-Net, and SNR-Net yield distorted reconstructions in both the sky and building regions. LEDNet cannot effectively recover overexposed areas and does not sufficiently enhance darker regions. EnlightenGAN introduces significant color shifts across the entire image. HVI-CIDNet generally underperforms in brightness recovery and struggles to capture the natural gradient of illumination. The fourth row focuses on the restoration of local details, indicated by red boxes. KinD, EnlightenGAN, and Retinex-Net produce unnatural enhancements and noticeable detail loss. Other methods also struggle with texture restoration, with SNR-Net and HVI-CIDNet exhibiting the relatively serious loss of fine structures. In comparison, the proposed method performs well in preserving and recovering local textures and structural details, demonstrating stable performance in terms of both visual fidelity and structural consistency.

In addition to subjective evaluation, we compare the proposed MSHCDI-Net against prevailing methods on LOL-v1 [17] and LOL-v2 [29]. PSNR and SSIM are employed to quantitatively assess the performance of the algorithms. As shown in Table 1, the proposed method achieves the best PSNR and SSIM on LOL-v1. For LOL-v2-real, it obtains the best SSIM and the second-best PSNR, while for LOL-v2-synthetic, it ranks second in both metrics. We can achieve the highest average PSNR and SSIM across the three datasets. This indicates that the proposed method provides more balanced and consistent performance under different low-light conditions. In addition, no-reference image quality metrics, namely Natural Image Quality Evaluator (NIQE) [30] and the Perceptual Image Quality Evaluator (PIQE) [31], are also adopted to further evaluate the perceptual quality of the enhanced images. As illustrated in Table 2, the proposed method continues to demonstrate competitive performance across these metrics. This balanced performance can be attributed to the proposed hybrid CNN–Transformer architecture and cross-domain feature fusion strategy, which jointly enhance local detail preservation and global illumination consistency.

**Table 1:** Quantitative comparison on LOL datasets. Optimal metrics are bolded, and suboptimal metrics are underlined.

Methods	LOL-v1		LOL-v2-real		LOL-v2-synthetic		Average	
	PSNR	SSIM	PSNR	SSIM	PSNR	SSIM	PSNR	SSIM
KinD[18]	20.86	0.790	14.74	0.641	13.29	0.578	16.29	0.669
EnlightenGAN [19]	17.48	0.650	15.31	0.538	18.31	0.608	17.03	0.598
Retinex-Net [17]	16.77	0.560	15.47	0.567	17.13	0.798	16.45	0.641
MIR-Net [14]	21.37	0.813	21.31	0.717	22.14	0.884	21.60	0.804
SNR-Net [24]	$\underset{―}{23.36}$	$\underset{―}{0.841}$	20.09	0.831	21.93	0.896	21.79	0.856
LLFormer [12]	22.82	0.834	19.03	0.740	22.37	0.876	21.40	0.816
LEDNet [28]	22.53	0.828	18.75	0.641	18.60	0.740	19.96	0.736
HVI-CIDNet [13]	22.22	0.833	**22.41**	$\underset{―}{0.848}$	**24.27**	**0.921**	$\underset{―}{22.97}$	$\underset{―}{0.867}$
Ours	**23.45**	**0.848**	$\underset{―}{22.24}$	**0.868**	$\underset{―}{23.74}$	$\underset{―}{0.910}$	**23.14**	**0.875**

**Table 2:** Evaluation of no-reference metrics. Optimal metrics are bolded, and suboptimal metrics are underlined.

Methods	LOL-v2-real		LOL-v2-synthetic		Average	
	NIQE	PIQE	NIQE	PIQE	NIQE	PIQE
KinD[18]	15.50	21.08	15.58	19.54	15.54	20.31
EnlightenGAN [19]	**9.69**	10.54	13.00	10.49	**11.35**	10.52
MIR-Net [14]	17.13	30.02	13.80	10.01	15.47	20.02
SNR-Net [24]	14.83	13.73	$\underset{―}{12.97}$	7.39	13.90	10.56
LLFormer [12]	16.89	$\underset{―}{5.43}$	13.66	**6.60**	15.25	$\underset{―}{6.02}$
LEDNet [28]	15.68	27.85	15.74	12.21	15.71	13.98
HVI-CIDNet [13]	14.86	7.09	**12.68**	9.49	13.77	8.29
Ours	$\underset{―}{11.35}$	**4.06**	13.44	$\underset{―}{7.36}$	$\underset{―}{12.40}$	**5.71**

To verify the effectiveness of the proposed MSHCDI-Net, we analyzed the average growth rates and reduction rates of key quantitative metrics (PSNR, SSIM, NIQE, PIQE), and the results are summarized in Table 3. Table 3 presents the relative improvement rates of MSHCDI-Net compared with all existing comparative methods, which are calculated based on the data obtained from Tables 1 and 2. The experimental results confirm the effectiveness of our proposed design: benefiting from the dual-branch architecture of MSHCDI-Net, it achieves obvious improvements compared with the traditional single-branch architecture. Specifically, it realizes a 42.05% increase in PSNR and a 30.79% increase in SSIM, as well as a 20.21% reduction in NIQE and a 71.88% reduction in PIQE, compared with KinD with a CNN architecture; compared with LLFormer with a Transformer architecture, it achieves an 8.13% increase in PSNR and a 7.23% increase in SSIM, as well as an 18.69% reduction in NIQE and a 5.15% reduction in PIQE; meanwhile, relying on the deep cross-domain feature fusion capability of MSHCDI-Net, it achieves a 6.20% increase in PSNR and a 2.22% increase in SSIM, as well as a 10.79% reduction in NIQE and a 45.93% reduction in PIQE, compared with SNR-Net. Although HVI-CIDNet shows strong performance on several full-reference metrics in Table 1; however, its no-reference results in Table 2 are not as competitive as those of MSHCDI-Net. In comparison, MSHCDI-Net achieves the best average PSNR and SSIM, as well as lower average NIQE and PIQE than HVI-CIDNet. This further suggests that the proposed method provides a more balanced performance in terms of reconstruction accuracy and perceptual quality.

**Table 3:** Relative improvement (%) of the proposed method compared with existing methods based on the average metrics reported in Tables 1 and 2. Positive values indicate improvement, while negative values indicate degradation.

Methods	PSNR ↑ (%)	SSIM ↑ (%)	NIQE ↓ (%)	PIQE ↓ (%)
KinD[18]	42.05	30.79	20.21	71.88
EnlightenGAN [19]	35.88	46.32	−9.25	45.72
MIR-Net [14]	7.13	8.83	19.84	71.48
SNR-Net [24]	6.20	2.22	10.79	45.93
LLFormer [12]	8.13	7.23	18.69	5.15
LEDNet [28]	15.93	18.89	43.04	59.16
HVI-CIDNet [13]	0.74	0.92	9.95	31.12

### 4.4 Ablation study of key components

The proposed framework comprises three key components: 1) Cross-Domain Feature Fusion (CFF) module, 2) Color Restoration (CR) module, and 3) Long-Distance Feature Extraction (LDFE) module. To verify the effectiveness of each key module, ablation experiments are conducted by removing one module at a time, and the results are presented in Table 4.

**Table 4:** Performance comparison of different models. Optimal metrics are bolded, and suboptimal metrics are underlined.

Model	PSNR	SSIM	NIQE	PIQE
w/o CFF	19.58	0.782	12.34	7.99
w/o CR	$\underset{―}{20.37}$	$\underset{―}{0.815}$	12.67	$\underset{―}{4.14}$
w/o LDEF	19.45	0.766	$\underset{―}{11.52}$	4.36
MSHCDI-Net	**22.24**	**0.848**	**11.35**	**4.06**

As shown in Table 4, the complete MSHCDI-Net achieves the optimal performance across all metrics (PSNR: 22.24, SSIM: 0.848, NIQE: 11.35, PIQE: 4.06), which is significantly superior to the variants with any single module removed. Specifically, the network realizes effective multi-scale feature interaction through the combined operation of the LDFE module (global) and the SDFE module (local); the removal of LDFE (w/o LDEF) leads to the lowest PSNR (19.45) and SSIM (0.766), confirming its critical role in global feature extraction. The CFF module effectively fuses global and local information, and its absence (w/o CFF) results in reduced PSNR (19.58) and SSIM (0.782), demonstrating the necessity of cross-domain fusion for feature complementarity. The CR module enhances low-light image quality through robust color restoration, and the variant without CR (w/o CR) shows worse color-related performance, as evidenced by the higher NIQE (12.67) compared to the complete model.

**Fig 13:**
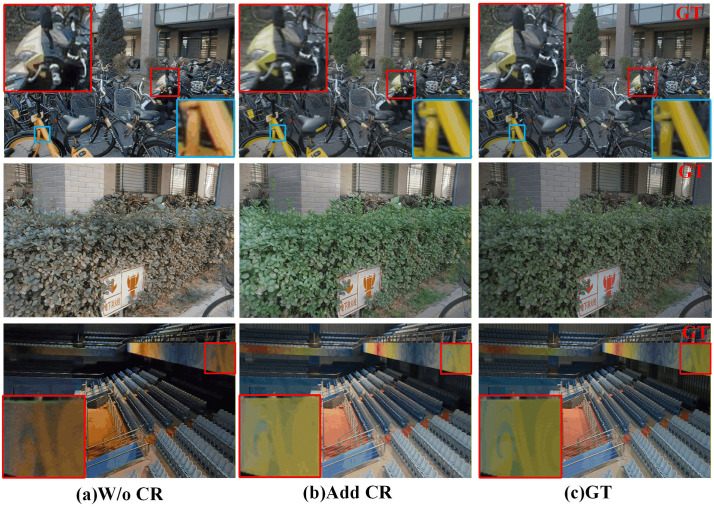
The comparison between the results with CR and without the CR module, as illustrated in the magnified regions, the model with CR enables a more effective preservation of the image’s color information.

Specifically, the CFF module captures and fuses fine-grained local details with a broader global context, which explains the performance degradation when it is removed. Meanwhile, the CR module recovers natural and accurate colors that are degraded under low-light conditions. As illustrated in Fig 13, the inclusion of the CR module results in markedly improved color restoration, which is particularly evident in the red-boxed regions encompassing the shared bicycle (blue box) and the electric bicycle. In the second row of the figure, the CR module enables the network to reconstruct more vivid and natural colors, which is consistent with the optimal PIQE (4.06) of the complete model in Table 4, further verifying the effectiveness of the CR module.

Furthermore, a systematic ablation study on the perceptual loss weight (λ) was conducted on the LOL-v2 dataset, with three settings: λ=0 (no perceptual loss), λ=1 (over-weighted), and λ=0.1 (final setting; results in Table 5). Unlike existing fixed-weight strategies, we determined the suitable λ=0.1 via ablation to balance objective metrics (PSNR/SSIM), subjective visual perception, and training stability, addressing the trade-off bias between objective fidelity and perceptual quality in prior work.

**Table 5:** Ablation study on perceptual loss weight on the LOL-v2 dataset. Optimal metrics are bolded, and suboptimal metrics are underlined.

Setting	PSNR	SSIM	NIQE	PIQE
λ=0 (w/o weighted)	20.18	0.827	12.28	4.23
λ=1 (over-weighted)	$\underset{―}{0.815}$	$\underset{―}{0.845}$	**10.95**	**4.05**
λ=0.1 (Final setting)	**22.24**	**0.868**	$\underset{―}{11.35}$	$\underset{―}{4.06}$

Experimental results show λ=0.1 achieves the best overall PSNR/SSIM performance, outperforming λ=0 and λ=1. While λ=1 improves no-reference perceptual metrics (lower NIQE/PIQE), it causes significant drops in PSNR/SSIM—attributed to overemphasis on visual texture perception at the expense of pixel-level fidelity, deviating from pixel reconstruction goals. Additionally, λ=1 leads to unstable training due to imbalanced loss function gradients, disrupting smooth convergence and final performance. This validates the rationality of λ=0.1, offering a reference for loss function design in LLIE. In summary, an appropriate perceptual loss weight λ=0.1 can balance the objective metrics and visual perception effect while ensuring stable training.

It should be noted that in Chapter 3 of this study, experiments such as feature visualization (Fig 6), attention map analysis (Fig 8), and convolutional kernel size optimization (Fig 10) are conducted to further verify the effectiveness and design rationality of the proposed modules. These experiments complement the ablation experiments in this section and jointly support the rationality and effectiveness of the proposed framework.

Beyond the ablation analysis of key components and loss function parameters, we further analyze the computational complexity and inference efficiency of the proposed model to verify its practicality. We attempt to balance efficiency and performance via a patch-based processing strategy while maintaining acceptable enhancement performance, aiming to alleviate the common issue that high-performance models tend to have high computational complexity and poor practicality.

To verify the practicality of the proposed model, we analyze its computational complexity and inference efficiency in detail. All experiments are conducted on a single NVIDIA RTX 4070 Ti GPU with 12 GB of video memory. A patch-based processing strategy is adopted, where the core patch size is set to 256×256 with an overlap of 200, to accommodate the input image size of 600×400. The proposed model contains 21.74 M trainable parameters, and the computational complexity for a 256×256 patch is 72.12 GFLOPs. Such a patch-based inference strategy effectively alleviates the efficiency pressure caused by the high computational load. Experimental results demonstrate that the average inference time for a single 600×400 low-light image is merely 0.3620 seconds. In summary, the model achieves a reasonable trade-off between performance, parameter scale, and computational complexity, which not only reflects the technical innovation of this work but also guarantees its practical application value. In future work, we will further optimize the lightweight design of the model to facilitate its wider deployment.

### 4.5 Generalization of the proposed method

**Fig 14:**
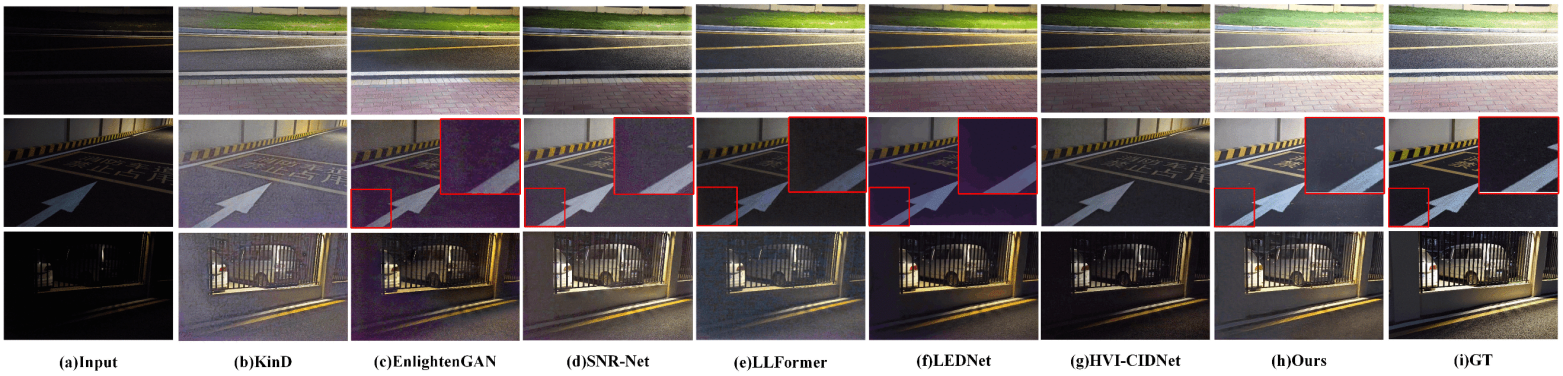
A comparison of generalization capability is presented by testing datasets from different domains. As illustrated in the enlarged region, the results of SNR-Net exhibit a significant amplification of noise. In contrast, the enhancement effects of LLFormer and LEDNet are not particularly pronounced, with the results appearing noticeably darker. The proposed algorithm, however, yields results that are much closer to the ground truth.

Data-driven algorithms typically exhibit a certain dependency on training samples. To evaluate the generalization capability of the proposed method, we directly tested on 83 randomly collected low-light images from natural scenes. The corresponding results are illustrated in Fig 14. In the first row, the output of KinD displays obvious noise, while ther comparative methods demonstrate various limitations. In the second row, evident purple artifacts can be observed along the edges in the results produced by EnlightenGAN, LLFormer, SNR-Net, and LEDNet, as indicated by the red boxes. Additionally, KinD and HVI-CIDNet suffer from insufficient denoising and inadequate brightness enhancement, respectively. In the third row, KinD, EnlightenGAN, and LLFormer produce noticeable noise and artifacts in the enhanced images, while LEDNet and HVI-CIDNet fail to adequately restore brightness. In contrast, the proposed method delivers stable enhancement performance across all tested scenes.

### 4.6 Limitation of the proposed framework

**Fig 15:**
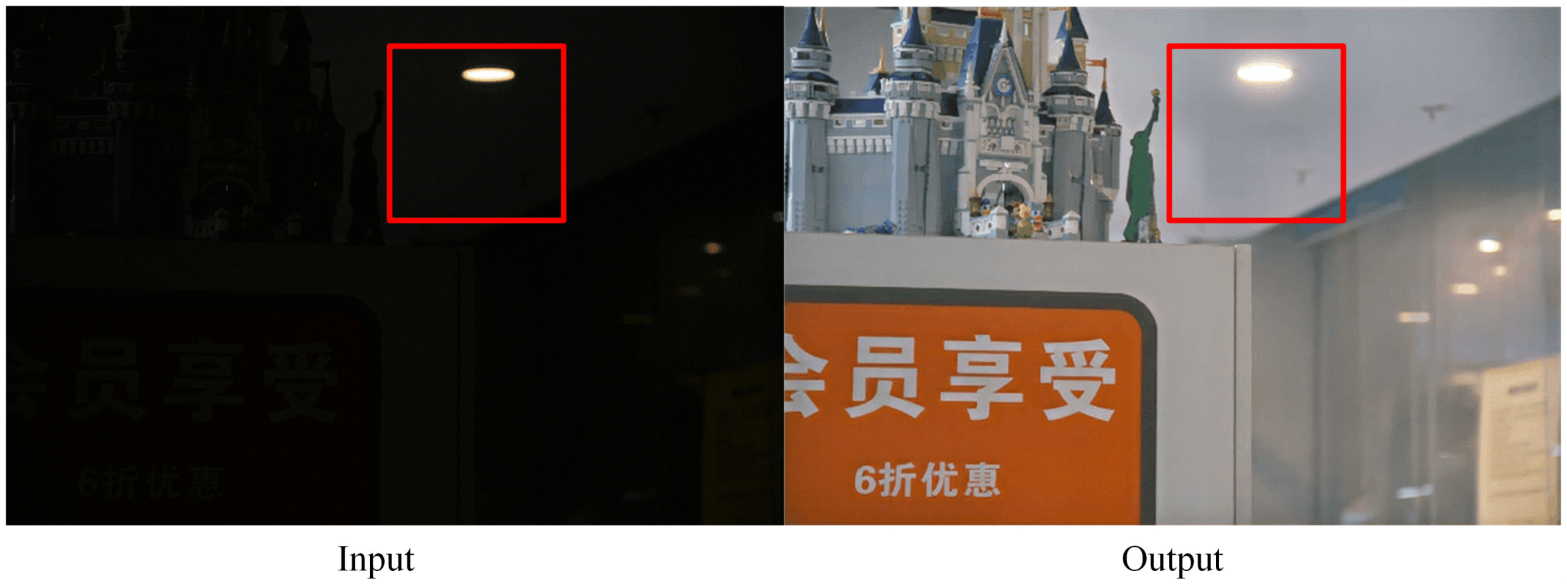
In the results presented in the figure, glow artifacts are clearly visible in the red – framed area, and there are also a small number of black artifacts around it.

The proposed method demonstrates clear advantages in contrast enhancement and detail preservation. However, glow artifacts are still present, as illustrated in Fig 15. This issue has been largely overlooked in the current low-light image enhancement (LLIE) literature. Notably, the artifacts may pose significant safety risks when LLIE techniques are deployed in safety-critical applications, such as autonomous robotic navigation and self-driving vehicles. Addressing this limitation will be a key focus of our future research.

## 5. Conclusion remarks and discussion

In this paper, a novel low-light image enhancement network is proposed, which integrates Convolutional Neural Networks (CNNs) with Transformer architectures. Specifically, MSHCDI-Net consists of a Short-Distance Feature Extraction (SDFE) module and a Long-Distance Feature Extraction (LDFE) module, enabling superior restoration of global and local illumination characteristics. Additionally, the Cross-Domain Feature Fusion (CFF) module reduces semantic discrepancies between the outputs of the two feature extraction modules through an iterative residual framework, thereby improving overall image quality. The Color Restoration (CR) module is also introduced to enhance color fidelity, resulting in more natural and visually pleasing outcomes. Experimental results show that MSHCDI-Net alleviates the limitations of traditional approaches in capturing long-range dependencies and recovering fine details by leveraging the strengths of both CNNs and Transformers.
