# Supplementary material for: Enhancing low-light images with MSHCDI-Net: A multi-scale hybrid cross-domain interaction approach
Source: PLoS One. 2026 Jul 17;21(7):e0352326. doi: 10.1371/journal.pone.0352326 (PMC13378987; doi:10.1371/journal.pone.0352326)
Supplement: Attachment — Submitted filename: Response Letter.pdf [file pone.0352326.s003.pdf]

Title: Enhancing Low-Light Images with MSHCDI-Net: A Multi-Scale Hybrid Cross-Domain Interaction Approach

Authors: Bin Chen, Peitao Li, Chaobing Zheng, Meng Jia, Shiqian Wu

We thank the editors and reviewers very much for their further efforts in handling this manuscript and providing many helpful feedbacks and constructive comments. Below, we address the questions and concerns raised by all of our reviewers. The reviewers' comments are in black, while our responses are in blue.

Replies to AE's Comments

1.Please ensure that your manuscript meets PLOS ONE's style requirements, including those for file naming. The PLOS ONE style templates can be found at https://journals.plos.org/plosone/s/file?id=wjVg/PLOSOne_formatting_sample_main_body.pdf and https://journals.plos.org/plosone/s/file?id=ba62/PLOSOne_formatting_sample_title_authors_affiliations.pdf

Replies: We have carefully reviewed the manuscript and checked it against the PLOS ONE style requirements, including formatting and file naming. Necessary revisions have been made to ensure full compliance with the journal's guidelines.

2.Please note that PLOS One has specific guidelines on code sharing for submissions in which author-generated code underpins the findings in the manuscript. In these cases, we expect all author-generated code to be made available without restrictions upon publication of the work. Please review our guidelines at https://journals.plos.org/plosone/s/materials-and-software-sharing#loc-sharing-code and ensure that your code is shared in a way that follows best practice and facilitates reproducibility and reuse.

Replies: The link to the code and data repository has been provided in the Abstract section of the manuscript.

3. Please ensure that you refer to Figure 13 in your text as, if accepted, production will need this reference to link the reader to the figure.

Replies: Thank you for the reminder. We have added an explicit reference to Figure 13 in the main text to ensure clear linkage between the text and the figure for production purposes.

4. We note that Figure(s) 1, 2, 6, 8, 9, 10, 11, 12, 13, 14, 15 in your submission contain copyrighted images. All PLOS content is published under the Creative Commons Attribution License (CC BY 4.0), which means that the manuscript, images, and Supporting Information files will be freely available online, and any third party is permitted to access, download, copy, distribute, and use these materials in any way, even commercially, with proper attribution. For more information, see our copyright guidelines: http://journals.plos.org/plosone/s/licenses-and-copyright.
Replies: Thank you for your reminder regarding the copyright issue.We have carefully checked and revised all the figures in our manuscript. We have revised Figure 1, Figure 11, Figure 12, and Figure 13, and removed all copyrighted images from these figures.All remaining figures are created and processed by our authors, using only our own processed images instead of the original copyrighted ones. We confirm that all figures in the revised manuscript comply with the CC BY 4.0 license of PLOS ONE, and no copyrighted materials are included.

5. We are unable to open your Supporting Information file [plos2025.bts, plos_bibtex_sample.bib, plos_latex_template.aux, plos_latex_template.bbl, plos_latex_template.blg, plos_latex_template.log, efd09db5-868f-445d-a5ae-f13bcc4f2015, plos_latex_template.tex,]. Please kindly revise as necessary and re-upload.
Replies: We apologize for the misunderstanding and the inconvenience caused. We initially misinterpreted the Supporting Information requirement and incorrectly uploaded some auxiliary LaTeX files. We have now removed these irrelevant files and uploaded only the correct and complete Supporting Information materials as required by the journal.

6. If the reviewer comments include a recommendation to cite specific previously published works, please review and evaluate these publications to determine whether they are relevant and should be cited. There is no requirement to cite these works unless the editor has indicated otherwise.

Replies: We have carefully checked all reviewer comments. No specific citation suggestions were provided in the reviews. We will continue to maintain appropriate and standard citation practices in accordance with the journal's guidelines.

Replies to Reviewer 1's Comments

Comments:

1.The novelty of this manuscript and the motivation is not clear. The authors should provide the framework figure of the proposed method.

Replies: We sincerely appreciate the reviewer's valuable and constructive comments. We have carefully revised the manuscript to clarify the motivation and novelty, and we have added and improved the framework figure as suggested.

1.Clarification of motivation and novelty:

We have thoroughly rewritten and strengthened the Introduction section to clearly point out the limitations of existing low-light image enhancement methods, including insufficient balance between local detail preservation and global illumination modeling, shallow cross-domain feature fusion in existing hybrid CNN-Transformer architectures, and severe color distortion. Based on these challenges, we clearly explain the research motivation and the novelty of the proposed MSHCDI-Net. The core innovations, including the dual-branch structure, the Cross-Domain Feature Fusion (CFF) module, and the Color Restoration (CR) module, are explicitly emphasized. All revisions are highlighted in red in the revised manuscript.

2. Presentation of the framework figure:

Figure 2 illustrates the overall framework of the proposed method. We have also explicitly referred to this framework figure in the Introduction section.

3. Contributions:

In the Introduction section, we clearly summarize the contributions of this paper as follows:

We propose a novel dual-branch Multi-Scale Hybrid Cross-Domain Interaction Network (MSHCDI-Net) for LLIE, which achieves a better trade-off between local detail preservation and global illumination consistency via multi-scale interaction, rather than simple CNN-Transformer integration.

We design a Cross-Domain Feature Fusion (CFF) module to enable deep, hierarchical, and adaptive interaction between CNN-based local features and Transformer-based global features, alleviating the shallow fusion problem in existing hybrid architectures.

We introduce a dedicated Color Restoration (CR) module to alleviate color distortion in LLIE, improving chromatic fidelity without extra computational cost.

Extensive experiments on both benchmark and real-world low-light datasets demonstrate that the proposed MSHCDI-Net achieves competitive visual and quantitative results with high efficiency, verifying the effectiveness and practicality of our design.

All modifications are clearly tracked in red text throughout the revised manuscript for your convenience. We believe these revisions substantially improve the clarity and rigor of this work.

2.Provide a discussion for why this Transposed self-attention mechanism is beneficial for low-light image enhancement compared to alternative non-local or window-based attention approaches.

Replies: We sincerely appreciate the reviewer's valuable suggestion. According to your comment, we have added a dedicated discussion in Section 4.2 of the revised manuscript (all revisions are highlighted in red) to explain why the Transposed self-attention mechanism is more suitable for low-light image enhancement, compared with vanilla self-attention, window-based attention, and non-local attention.

Specifically, we have supplemented explanations from the following three aspects:

Computational efficiency: We clarify the computational advantage of Transposed self-attention over vanilla self-attention, especially when processing high-resolution low-light images.

Local-global feature synergy: We explain how Transposed self-attention achieves better balance between global illumination consistency and local detail preservation, compared with window-based attention.

Attention stability: We analyze how Transposed self-attention suppresses noise amplification and improves robustness, in contrast to non-local attention.

All the above revisions are clearly marked in red in Section 4.2. We believe these additions fully address your concern and further improve the rationality and rigor of our method.

3.Include an ablation study or discussion to justify the choice of the weights of loss function.

Replies: We sincerely appreciate the reviewer's constructive suggestion. To justify the choice of the weights in our loss function, we have added a systematic ablation study and detailed discussion in **Section 5.4 "Ablation Study of Key Components"** of the revised manuscript (all revisions are highlighted in red). A new Table 5 has also been added to clearly present the experimental results of this ablation study.

We analyzed the performance differences under different weight settings, clarified the drawbacks of under-weighted (λ=0) and over-weighted (λ=1) perceptual loss, and verified that our adopted weight (λ=0.1) achieves the best balance between performance and training stability.

Replies to Reviewer 2's Comments

Comments:

1. Insufficient novelty and contribution.

Replies: We sincerely appreciate the reviewer's critical feedback on the novelty and contribution of our work. To address the concern of incremental contribution and limited theoretical innovation, we have comprehensively revised the Introduction and Related Works sections (highlighted in red) to strengthen the novelty justification and clarify the unique value of our proposed MSHCDI-Net relative to existing hybrid CNN-Transformer models. Key revisions are summarized as follows:

Refined problem formulation to highlight unmet challenges. We explicitly emphasize three critical and intertwined challenges in current LLIE research that have not been fully addressed by existing hybrid models: (1) the insufficient balance between local detail preservation and global illumination consistency; (2) shallow cross-domain feature interaction in CNN-Transformer fusion; (3) prevalent color distortion in low-light enhancement (a long-overlooked issue in hybrid architectures). This reframing clearly positions our work to solve unaddressed gaps rather than making incremental improvements.

Strengthened novelty of the proposed framework ,We revised the description of MSHCDI-Net to emphasize that our dual-branch architecture transcends simple CNN-Transformer concatenation (a common practice in existing hybrid models) and achieves deep, multi-scale cross-domain interaction. Specifically: The novel Cross-Domain Feature Fusion (CFF) module enables hierarchical, adaptive

interaction between CNN-local and Transformer-global features (not just one-way fusion), addressing the core limitation of shallow fusion in state-of-the-art hybrid models (e.g., HVI-CIDNet, SNRNet). The dedicated Color Restoration (CR) module is designed to mitigate color distortion without extra computational cost—this is a targeted innovation that supplements the color preservation capability missing in most existing hybrid LLIE models.The above content corresponds to the seventh paragraph of the revised Introduction section.

Enhanced the presentation of contributions to avoid overstatement and highlight uniqueness:

1A novel hybrid framework, MSHCDI-Net, is presented for low-light image enhancement, aiming to address the limited interaction between CNN-based local representations and Transformer-derived global contexts in existing hybrid architectures. A Cross-Domain Feature Fusion (CFF) module is introduced to enable deep, hierarchical, and adaptive cross-domain feature interaction, facilitating effective information exchange and strengthening complementary representation learning.

2 A lightweight Color Restoration (CR) module is further developed to alleviate color distortion commonly observed in low-light enhancement. The module enhances chromatic consistency and color fidelity while introducing negligible computational overhead, thereby improving visual realism and complementing the limitations of current methods.

All revisions are highlighted in red in the Introduction (Section 1) and Related Works (Section 2) for the reviewer's convenience. We hope these improvements can better clarify the motivation and novelty of our work, and help distinguish it from existing hybrid models for low-light image enhancement.

2. Absence of computational complexity and runtime analysis.

Replies: We sincerely appreciate the reviewer's valuable suggestion regarding computational complexity and runtime analysis, which helps improve the experimental rigor and practicality of our work. To address this concern, we have added detailed analysis of computational complexity and inference efficiency in Section 5.3 "Comparisons with Different Algorithms" of the revised manuscript (highlighted in red).

Specifically, we conducted all efficiency tests on a single NVIDIA RTX 4070 Ti GPU (12GB VRAM), adopting a core 256×256 patch-based processing strategy (with

an overlap size of 200) to adapt to the 600×400 input image size. We first report the model's basic efficiency metrics: the total number of trainable parameters of the proposed MSHCDI-Net is 21.74 M, and the computational complexity corresponding to a 256×256 patch is 72.12 G FLOPs.

To mitigate the common issue that high-performance models often suffer from high computational complexity and poor practicality, we innovatively applied a patch-based processing strategy, which effectively alleviates the efficiency pressure caused by high computational load. Experimental results show that the average inference time for a single 600×400 low-light image is only 0.3620 seconds.

3. Efficiency comparisons

Replies: Good advice. To further improve the experimental rigor and provide more convincing evidence, we have added efficiency comparisons in Section 5.3 "Comparisons with Different Algorithms" (all revisions are highlighted in red).

Specifically, we have added a new Table 3 to present the relative improvement percentages of our MSHCDI-Net over state-of-the-art methods in terms of PSNR, SSIM, NIQE, and PIQE. Based on the average metric values from Tables 1 and 2, we quantitatively evaluate the performance gains of the proposed method in a unified and comparable manner. Such statistical analysis demonstrates the stability and superiority of our method in a more intuitive and convincing way, which enhances the statistical robustness of the experimental results.

Table 3. Relative improvement (%) of the proposed method compared with existing methods based on the average metrics reported in Tables 1 and 2. Positive values indicate improvement, while negative values indicate degradation.

Methods	PSNR ↑ (%)	SSIM ↑ (%)	NIQE ↓ (%)	PIQE ↓ (%)
KIND	42.05	30.79	20.21	71.88
EnlightenGAN	35.88	46.32	-9.25	45.72
MIR-Net	7.13	8.83	19.84	71.48
SNR-Net	6.20	2.22	10.79	45.93
LLFormer	8.13	7.23	18.69	5.15
LEDNet	15.93	18.89	43.04	59.16
HVI-CIDNet	0.74	0.92	9.95	31.12

4. Limited depth in ablation studies.

Replies: we have substantially revised and extended the ablation study section, with all revisions highlighted in red. Specifically, we have added an additional ablation experiment on the loss function weight to justify the rationality of our loss function

design. Meanwhile, we have supplemented the computational complexity and inference efficiency analysis to verify the practicality of the proposed model.

5.Some overstatements in claims

Replies: We sincerely appreciate the reviewer's careful reminder regarding potential overstatements in the manuscript. We have carefully rechecked and revised the entire manuscript to adjust inappropriate expressions. All exaggerated or inappropriate descriptions have been modified to ensure the language is more rigorous, objective, and modest. We have avoided overstatement and strengthened the preciseness of the presentation throughout the paper.

Replies to Reviewer 3' s Comments

Comments:

This research paper " Enhancing Low-Light images with Multi-Scale Hybrid Cross-Domain Interaction Approach" proposes a novel hybrid deep-learning architecture 'MSHCDI-Net' that integrates Convolutional Neural Networks (CNNs) and Transformers for low-light image enhancement (LLIE). The paper is well written.

Replies: We sincerely appreciate Reviewer for your comments and recognition of our manuscript. We are delighted to learn that our paper is considered well-written, which encourages us greatly. We have carefully considered all the suggestions from the reviewers to further improve the quality of our work, and we hope the revised manuscript meets the publication standards.
