# Supplementary material for: Enhancing low-light images with MSHCDI-Net: A multi-scale hybrid cross-domain interaction approach
Source: PLoS One. 2026 Jul 17;21(7):e0352326. doi: 10.1371/journal.pone.0352326 (PMC13378987; doi:10.1371/journal.pone.0352326)
Supplement: Attachment — Submitted filename: Response Letter_20260521162457.pdf [file pone.0352326.s004.pdf]

Title: Enhancing Low-Light Images with MSHCDI-Net: A Multi-Scale Hybrid Cross-Domain Interaction Approach

Authors: Bin Chen, Peitao Li, Chaobing Zheng, Meng Jia, Shiqian Wu

We thank the editors and reviewers very much for their further efforts in handling this manuscript and providing many helpful feedbacks and constructive comments. Below, we address the questions and concerns raised by all of our reviewers. The reviewers' comments are in black, while our responses are in blue.

Replies to AE's Comments

1. The reviewers have provided several comments and suggestions regarding the manuscript. In particular, clarification is required for the comparisons presented in Table 1, including the selection of existing models used for evaluation. Additionally, the claims regarding the superiority and effectiveness of the proposed model require further justification.

Replies: We sincerely thank the AE and the reviewers for their valuable comments. These suggestions have played a crucial role in improving the quality and clarity of our manuscript. We have clarified the rationale for selecting the compared methods in Table 1 and revised the discussion of the proposed model's performance to avoid overgeneralized claims of superiority. The detailed changes are described in the responses to Reviewer #4 and Reviewer #5 below.

Replies to Reviewer 4's Comments

Comments:

1. The manuscript contains minor issues related to justification and typographical errors that should be addressed.

Replies: We sincerely apologize for making such mistakes. We have carefully gone through the entire manuscript again and corrected the typographical errors and formatting issues accordingly.

2. In Table 1, the comparison between the proposed model and existing models requires further clarification. Some of the referenced models are from conference publications, while others are not recent. The authors are requested to justify the selection of these models for comparison.

Replies: We sincerely thank the reviewer for this valuable comment. In the revised manuscript, we have further clarified the rationale for selecting the comparison methods in Section 5.2.These methods cover the main LLIE paradigms. Specifically, Retinex-Net, KinD, MIRNet, and SNR-Net are CNN-based approaches; EnlightenGAN is a GAN-based method; LLFormer is a Transformer-based model; and SNR-Net and HVI-CIDNet further represent hybrid architectures combining CNN and Transformer components.This selection enables a comprehensive evaluation across different design philosophies, facilitating a balanced and fair comparison with state-of-the-art LLIE methods.

3. Additionally, the proposed model does not demonstrate superior performance across all aspects. The authors are therefore requested to provide adequate justification for these claims, particularly with reference to Table 1.

Replies: We sincerely thank the reviewer for this insightful comment. We agree that the proposed method does not achieve the best performance on every individual metric. In the revised manuscript, we have clarified this point and provided a more balanced discussion with respect to Table 1. Specifically, the proposed model is designed to achieve an overall trade-off between enhancement quality and visual naturalness, rather than optimizing a single metric in isolation.

Replies to Reviewer 5's Comments

Comments:

1.In Section 5, under 5.1 subsection there is a heading of Dataset. But, the description of it is missing.

Replies: Thank you for pointing this out. We have added a dataset description in Section 5.1, introducing LOL-v1, LOL-v2-real, and LOL-v2-synthetic and explaining their roles in evaluating the performance, robustness, and generalization ability of the proposed method.

2. In some places, there is write-up error, e.g., under 4.6 subsection, the "does not" is used twice. Please check.

Rplies: Very sorry for making such mistakes, we have carefully reviewed the entire manuscript and corrected identified issues.

3. Keep consistent wording in entire paper, e.g., KinD or KIND.

Replies: Thank you for the suggestion. We have checked the entire manuscript and unified the naming of this method as "KinD" throughout the paper.

4. Please check the alignments of words, space and other grammatical.

Replies: Thank you for this comment. We have carefully checked the manuscript and corrected spacing, punctuation, alignment, and grammatical issues.
