# Supplementary material for: Enhancing low-light images with MSHCDI-Net: A multi-scale hybrid cross-domain interaction approach
Source: PLoS One. 2026 Jul 17;21(7):e0352326. doi: 10.1371/journal.pone.0352326 (PMC13378987; doi:10.1371/journal.pone.0352326)
Supplement: Attachment — Submitted filename: PLOP.docx [file pone.0352326.s002.docx]

I appreciate the authors’ effort in preparing the manuscript; however, the incorporation of the following comments is recommended before its final publication.

1. The manuscript contains minor issues related to justification and typographical errors that should be addressed.
2. In Table 1, the comparison between the proposed model and existing models requires further clarification. Some of the referenced models are from conference publications, while others are not recent. The authors are requested to justify the selection of these models for comparison.
3. Additionally, the proposed model does not demonstrate superior performance across all aspects. The authors are therefore requested to provide adequate justification for these claims, particularly with reference to Table 1.
