# Supplementary material for: Enhancing low-light images with MSHCDI-Net: A multi-scale hybrid cross-domain interaction approach
Source: PLoS One. 2026 Jul 17;21(7):e0352326. doi: 10.1371/journal.pone.0352326 (PMC13378987; doi:10.1371/journal.pone.0352326)
Supplement: Peer review history

# Peer review: Decision Letter 0

**DOI:** 10.1371/journal.pone.0352326.r001

**Authors:** Nagaraju Y

3 Mar 2026

PONE-D-25-60023

Enhancing Low-Light Images with MSHCDI-Net: A Multi-Scale Hybrid Cross-Domain Interaction Approach

PLOS One

Dear Dr. Zheng,

Thank you for submitting your manuscript to PLOS ONE. After careful consideration, we feel that it has merit but does not fully meet PLOS ONE’s publication criteria as it currently stands. Therefore, we invite you to submit a **major revised version**  of the manuscript that addresses the points raised during the review process.

You are required to address all concerns raised by the reviewers and ensure that the image quality complies with the journal’s publication standards. Manuscripts that fail to meet these standards will be rejected without further consideration.

Please submit your revised manuscript by Apr 17 2026 11:59PM. If you will need more time than this to complete your revisions, please reply to this message or contact the journal office at plosone@plos.org. When you're ready to submit your revision, log on to https://www.editorialmanager.com/pone/ and select the 'Submissions Needing Revision' folder to locate your manuscript file.

Please include the following items when submitting your revised manuscript:

- A letter that responds to each point raised by the academic editor and reviewer(s). You should upload this letter as a separate file labeled 'Response to Reviewers'.
- A marked-up copy of your manuscript that highlights changes made to the original version. You should upload this as a separate file labeled 'Revised Manuscript with Track Changes'.
- An unmarked version of your revised paper without tracked changes. You should upload this as a separate file labeled 'Manuscript'.

If you would like to make changes to your financial disclosure, please include your updated statement in your cover letter. Guidelines for resubmitting your figure files are available below the reviewer comments at the end of this letter.

If applicable, we recommend that you deposit your laboratory protocols in protocols.io to enhance the reproducibility of your results. Protocols.io assigns your protocol its own identifier (DOI) so that it can be cited independently in the future. For instructions see: https://journals.plos.org/plosone/s/submission-guidelines#loc-laboratory-protocols. Additionally, PLOS ONE offers an option for publishing peer-reviewed Lab Protocol articles, which describe protocols hosted on protocols.io. Read more information on sharing protocols at https://plos.org/protocols?utm_medium=editorial-email&utm_source=authorletters&utm_campaign=protocols.

We look forward to receiving your revised manuscript.

Kind regards,

Nagaraju Y, Ph.D.

Guest Editor

PLOS One

Journal Requirements:

When submitting your revision, we need you to address these additional requirements.

1. Please ensure that your manuscript meets PLOS ONE's style requirements, including those for file naming. The PLOS ONE style templates can be found at

https://journals.plos.org/plosone/s/file?id=wjVg/PLOSOne_formatting_sample_main_body.pdf and https://journals.plos.org/plosone/s/file?id=ba62/PLOSOne_formatting_sample_title_authors_affiliations.pdf

2. Please note that PLOS One has specific guidelines on code sharing for submissions in which author-generated code underpins the findings in the manuscript. In these cases, we expect all author-generated code to be made available without restrictions upon publication of the work. Please review our guidelines at https://journals.plos.org/plosone/s/materials-and-software-sharing#loc-sharing-code and ensure that your code is shared in a way that follows best practice and facilitates reproducibility and reuse.

3. Please ensure that you refer to Figure 13 in your text as, if accepted, production will need this reference to link the reader to the figure.

4. We note that Figure(s) 1, 2, 6, 8, 9, 10, 11, 12, 13, 14, 15 in your submission contain copyrighted images. All PLOS content is published under the Creative Commons Attribution License (CC BY 4.0), which means that the manuscript, images, and Supporting Information files will be freely available online, and any third party is permitted to access, download, copy, distribute, and use these materials in any way, even commercially, with proper attribution. For more information, see our copyright guidelines: http://journals.plos.org/plosone/s/licenses-and-copyright.

We require you to either (1) present written permission from the copyright holder to publish these figures specifically under the CC BY 4.0 license, or (2) remove the figures from your submission:

a. You may seek permission from the original copyright holder of Figure(s) 1, 2, 6, 8, 9, 10, 11, 12, 13, 14, 15 to publish the content specifically under the CC BY 4.0 license.

We recommend that you contact the original copyright holder with the Content Permission Form (http://journals.plos.org/plosone/s/file?id=7c09/content-permission-form.pdf) and the following text:

“I request permission for the open-access journal PLOS ONE to publish XXX under the Creative Commons Attribution License (CCAL) CC BY 4.0 (http://creativecommons.org/licenses/by/4.0/). Please be aware that this license allows unrestricted use and distribution, even commercially, by third parties. Please reply and provide explicit written permission to publish XXX under a CC BY license and complete the attached form.”

Please upload the completed Content Permission Form or other proof of granted permissions as an "Other" file with your submission.

In the figure caption of the copyrighted figure, please include the following text: “Reprinted from [ref] under a CC BY license, with permission from [name of publisher], original copyright [original copyright year].”

b. If you are unable to obtain permission from the original copyright holder to publish these figures under the CC BY 4.0 license or if the copyright holder’s requirements are incompatible with the CC BY 4.0 license, please either i) remove the figure or ii) supply a replacement figure that complies with the CC BY 4.0 license. Please check copyright information on all replacement figures and update the figure caption with source information. If applicable, please specify in the figure caption text when a figure is similar but not identical to the original image and is therefore for illustrative purposes only.

5. We are unable to open your Supporting Information file [plos2025.bts, plos_bibtex_sample.bib, plos_template.aux, plos_template.bbl, plos_template.blg, plos_template.log, efd09db5-868f-445d-a5ae-f13bcc4f2015, plos_template.tex, ]. Please kindly revise as necessary and re-upload.

6. If the reviewer comments include a recommendation to cite specific previously published works, please review and evaluate these publications to determine whether they are relevant and should be cited. There is no requirement to cite these works unless the editor has indicated otherwise.

[Note: HTML markup is below. Please do not edit.]

Reviewers' comments:

Reviewer's Responses to Questions

**Comments to the Author**

1. Is the manuscript technically sound, and do the data support the conclusions?

The manuscript must describe a technically sound piece of scientific research with data that supports the conclusions. Experiments must have been conducted rigorously, with appropriate controls, replication, and sample sizes. The conclusions must be drawn appropriately based on the data presented.

Reviewer #1: Yes

Reviewer #2: Partly

Reviewer #3: Yes

**********

2. Has the statistical analysis been performed appropriately and rigorously?

Reviewer #1: Yes

Reviewer #2: I Don't Know

Reviewer #3: Yes

**********

3. Have the authors made all data underlying the findings in their manuscript fully available?

The PLOS Data policy requires authors to make all data underlying the findings described in their manuscript fully available without restriction, with rare exception (please refer to the Data Availability Statement in the manuscript PDF file). The data should be provided as part of the manuscript or its supporting information, or deposited to a public repository. For example, in addition to summary statistics, the data points behind means, medians and variance measures should be available. If there are restrictions on publicly sharing data—e.g. participant privacy or use of data from a third party—those must be specified.

Reviewer #1: Yes

Reviewer #2: Yes

Reviewer #3: Yes

**********

4. Is the manuscript presented in an intelligible fashion and written in standard English?

PLOS ONE does not copyedit accepted manuscripts, so the language in submitted articles must be clear, correct, and unambiguous. Any typographical or grammatical errors should be corrected at revision, so please note any specific errors here.

Reviewer #1: Yes

Reviewer #2: Yes

Reviewer #3: Yes

**********

5. Review Comments to the Author

Please use the space provided to explain your answers to the questions above. You may also include additional comments for the author, including concerns about dual publication, research ethics, or publication ethics. (Please upload your review as an attachment if it exceeds 20,000 characters)

Reviewer #1: This manuscript present a multi-scale hybrid cross-domain interaction network for low-light image enhancement, which integrates SDFE for local texture refinement , LDFE for global illumination modeling , CFF for hierarchical cross-domain feature interaction , and CR for high-quality color recovery. Experimental results show superior performance compared to state-of-the-art methods on the LOL datasets. However, there are some concerns:

1.The novelty of this manuscript and the motivation is not clear. The authors should provide the framework figure of the proposed method.

2.Provide a discussion for why this Transposed self-attention mechanism is beneficial for low-light image enhancement compared to alternative non-local or window-based attention approaches.

3.Include an ablation study or discussion to justify the choice of the weights of loss function.

Reviewer #2: The manuscript presents a hybrid CNN–Transformer architecture (MSHCDI-Net) for low-light image enhancement that demonstrates competitive performance on LOL datasets and includes ablation studies and both reference and no-reference evaluations; however, the contribution is largely incremental relative to existing hybrid models, with limited theoretical innovation, modest performance margins, and insufficient experimental rigor. Key weaknesses include lack of statistical validation, absence of computational complexity and runtime analysis, limited depth in ablation studies, and some overstatements in claims, along with writing and formatting issues that require polishing. While the work fits the journal’s scope and shows solid engineering implementation, it is not publication-ready in its current form and would require substantial revision—particularly strengthening the novelty justification, adding efficiency comparisons and statistical robustness, and improving clarity—to be suitable for acceptance.

Reviewer #3: This research paper " Enhancing Low-Light images with Multi-Scale Hybrid Cross-Domain Interaction Approach" proposes a novel hybrid deep-learning architecture 'MSHCDI-Net' that integrates Convolutional Neural Networks (CNNs) and Transformers for low-light image enhancement (LLIE). The paper is well written.

**********

6. PLOS authors have the option to publish the peer review history of their article (what does this mean?). If published, this will include your full peer review and any attached files.

If you choose “no”, your identity will remain anonymous but your review may still be made public.

**Do you want your identity to be public for this peer review?**  For information about this choice, including consent withdrawal, please see our Privacy Policy.

Reviewer #1: No

Reviewer #2: No

Reviewer #3: **Yes:** SAROJINI BALAKRISHNAN

**********

[NOTE: If reviewer comments were submitted as an attachment file, they will be attached to this email and accessible via the submission site. Please log into your account, locate the manuscript record, and check for the action link "View Attachments". If this link does not appear, there are no attachment files.]

To ensure your figures meet our technical requirements, please review our figure guidelines: https://journals.plos.org/plosone/s/figures

You may also use PLOS’s free figure tool, NAAS, to help you prepare publication quality figures: https://journals.plos.org/plosone/s/figures#loc-tools-for-figure-preparation.

NAAS will assess whether your figures meet our technical requirements by comparing each figure against our figure specifications.

# Author comment: Author response to Decision Letter 1

**DOI:** 10.1371/journal.pone.0352326.r002

24 Mar 2026

Replies to Reviewer 1’s Comments

Comments:

1.The novelty of this manuscript and the motivation is not clear. The authors should provide the framework figure of the proposed method.

Replies: We sincerely appreciate the reviewer’s valuable and constructive comments. We have carefully revised the manuscript to clarify the motivation and novelty, and we have added and improved the framework figure as suggested.

1.Clarification of motivation and novelty:

We have thoroughly rewritten and strengthened the Introduction section to clearly point out the limitations of existing low-light image enhancement methods, including insufficient balance between local detail preservation and global illumination modeling, shallow cross-domain feature fusion in existing hybrid CNN-Transformer architectures, and severe color distortion. Based on these challenges, we clearly explain the research motivation and the novelty of the proposed MSHCDI-Net. The core innovations, including the dual-branch structure, the Cross-Domain Feature Fusion (CFF) module, and the Color Restoration (CR) module, are explicitly emphasized. All revisions are highlighted in red in the revised manuscript.

2. Presentation of the framework figure:

Figure 2 illustrates the overall framework of the proposed method. We have also explicitly referred to this framework figure in the Introduction section.

3. Contributions:

In the Introduction section, we clearly summarize the contributions of this paper as follows:

We propose a novel dual-branch Multi-Scale Hybrid Cross-Domain Interaction Network (MSHCDI-Net) for LLIE, which achieves a better trade-off between local detail preservation and global illumination consistency via multi-scale interaction, rather than simple CNN-Transformer integration.

We design a Cross-Domain Feature Fusion (CFF) module to enable deep, hierarchical, and adaptive interaction between CNN-based local features and Transformer-based global features, alleviating the shallow fusion problem in existing hybrid architectures.

We introduce a dedicated Color Restoration (CR) module to alleviate color distortion in LLIE, improving chromatic fidelity without extra computational cost.

Extensive experiments on both benchmark and real-world low-light datasets demonstrate that the proposed MSHCDI-Net achieves competitive visual and quantitative results with high efficiency, verifying the effectiveness and practicality of our design.

All modifications are clearly tracked in red text throughout the revised manuscript for your convenience. We believe these revisions substantially improve the clarity and rigor of this work.

2.Provide a discussion for why this Transposed self-attention mechanism is beneficial for low-light image enhancement compared to alternative non-local or window-based attention approaches.

Replies: We sincerely appreciate the reviewer’s valuable suggestion. According to your comment, we have added a dedicated discussion in Section 4.2 of the revised manuscript (all revisions are highlighted in red) to explain why the Transposed self-attention mechanism is more suitable for low-light image enhancement, compared with vanilla self-attention, window-based attention, and non-local attention.

Specifically, we have supplemented explanations from the following three aspects:

Computational efficiency: We clarify the computational advantage of Transposed self-attention over vanilla self-attention, especially when processing high-resolution low-light images.

Local-global feature synergy: We explain how Transposed self-attention achieves better balance between global illumination consistency and local detail preservation, compared with window-based attention.

Attention stability: We analyze how Transposed self-attention suppresses noise amplification and improves robustness, in contrast to non-local attention.

All the above revisions are clearly marked in red in Section 4.2. We believe these additions fully address your concern and further improve the rationality and rigor of our method.

3.Include an ablation study or discussion to justify the choice of the weights of loss function.

Replies: We sincerely appreciate the reviewer’s constructive suggestion. To justify the choice of the weights in our loss function, we have added a systematic ablation study and detailed discussion in Section 5.4 “Ablation Study of Key Components” of the revised manuscript (all revisions are highlighted in red). A new Table 5 has also been added to clearly present the experimental results of this ablation study.

We analyzed the performance differences under different weight settings, clarified the drawbacks of under-weighted (λ=0) and over-weighted (λ=1) perceptual loss, and verified that our adopted weight (λ=0.1) achieves the best balance between performance and training stability.

Replies to Reviewer 2’s Comments

Comments:

1.Insufficient novelty and contribution.

Replies: We sincerely appreciate the reviewer’s critical feedback on the novelty and contribution of our work. To address the concern of incremental contribution and limited theoretical innovation, we have comprehensively revised the Introduction and Related Works sections (highlighted in red) to strengthen the novelty justification and clarify the unique value of our proposed MSHCDI-Net relative to existing hybrid CNN-Transformer models. Key revisions are summarized as follows:

Refined problem formulation to highlight unmet challenges. We explicitly emphasize three critical and intertwined challenges in current LLIE research that have not been fully addressed by existing hybrid models: (1) the insufficient balance between local detail preservation and global illumination consistency; (2) shallow cross-domain feature interaction in CNN-Transformer fusion; (3) prevalent color distortion in low-light enhancement (a long-overlooked issue in hybrid architectures). This reframing clearly positions our work to solve unaddressed gaps rather than making incremental improvements.

Strengthened novelty of the proposed framework ,We revised the description of MSHCDI-Net to emphasize that our dual-branch architecture transcends simple CNN-Transformer concatenation (a common practice in existing hybrid models) and achieves deep, multi-scale cross-domain interaction. Specifically: The novel Cross-Domain Feature Fusion (CFF) module enables hierarchical, adaptive interaction between CNN-local and Transformer-global features (not just one-way fusion), addressing the core limitation of shallow fusion in state-of-the-art hybrid models (e.g., HVI-CIDNet, SNRNet). The dedicated Color Restoration (CR) module is designed to mitigate color distortion without extra computational cost—this is a targeted innovation that supplements the color preservation capability missing in most existing hybrid LLIE models.The above content corresponds to the seventh paragraph of the revised Introduction section.

Enhanced the presentation of contributions to avoid overstatement and highlight uniqueness:

1A novel hybrid framework, MSHCDI-Net, is presented for low-light image enhancement, aiming to address the limited interaction between CNN-based local representations and Transformer-derived global contexts in existing hybrid architectures. A Cross-Domain Feature Fusion (CFF) module is introduced to enable deep, hierarchical, and adaptive cross-domain feature interaction, facilitating effective information exchange and strengthening complementary representation learning.

2 A lightweight Color Restoration (CR) module is further developed to alleviate color distortion commonly observed in low-light enhancement. The module enhances chromatic consistency and color fidelity while introducing negligible computational overhead, thereby improving visual realism and complementing the limitations of current methods.

All revisions are highlighted in red in the Introduction (Section 1) and Related Works (Section 2) for the reviewer’s convenience. We hope these improvements can better clarify the motivation and novelty of our work, and help distinguish it from existing hybrid models for low-light image enhancement.

2. Absence of computational complexity and runtime analysis.

Replies: We sincerely appreciate the reviewer’s valuable suggestion regarding computational complexity and runtime analysis, which helps improve the experimental rigor and practicality of our work. To address this concern, we have added detailed analysis of computational complexity and inference efficiency in Section 5.3 “Comparisons with Different Algorithms” of the revised manuscript (highlighted in red).

Specifically, we conducted all efficiency tests on a single NVIDIA RTX 4070 Ti GPU (12GB VRAM), adopting a core 256×256 patch-based processing strategy (with an overlap size of 200) to adapt to the 600×400 input image size. We first report the model’s basic efficiency metrics: the total number of trainable parameters of the proposed MSHCDI-Net is 21.74 M, and the computational complexity corresponding to a 256×256 patch is 72.12 G FLOPs.

To mitigate the common issue that high-performance models often suffer from high computational complexity and poor practicality, we innovatively applied a patch-based processing strategy, which effectively alleviates the efficiency pressure caused by high computational load. Experimental results show that the average inference time for a single 600×400 low-light image is only 0.3620 seconds.

3. Efficiency comparisons

Replies: Good advice. To further improve the experimental rigor and provide more convincing evidence, we have added efficiency comparisons in Section 5.3 “Comparisons with Different Algorithms” (all revisions are highlighted in red).

Specifically, we have added a new Table 3 to present the relative improvement percentages of our MSHCDI-Net over state-of-the-art methods in terms of PSNR, SSIM, NIQE, and PIQE. Based on the average metric values from Tables 1 and 2, we quantitatively evaluate the performance gains of the proposed method in a unified and comparable manner. Such statistical analysis demonstrates the stability and superiority of our method in a more intuitive and convincing way, which enhances the statistical robustness of the experimental results.

4. Limited depth in ablation studies.

Replies: we have substantially revised and extended the ablation study section, with all revisions highlighted in red. Specifically, we have added an additional ablation experiment on the loss function weight to justify the rationality of our loss function design. Meanwhile, we have supplemented the computational complexity and inference efficiency analysis to verify the practicality of the proposed model.

5.Some overstatements in claims

Replies: We sincerely appreciate the reviewer’s careful reminder regarding potential overstatements in the manuscript. We have carefully rechecked and revised the entire manuscript to adjust inappropriate expressions. All exaggerated or inappropriate descriptions have been modified to ensure the language is more rigorous, objective, and modest. We have avoided overstatement and strengthened the preciseness of the presentation throughout the paper.

Replies to Reviewer 3’ s Comments

Comments:

This research paper " Enhancing Low-Light images with Multi-Scale Hybrid Cross-Domain Interaction Approach" proposes a novel hybrid deep-learning architecture 'MSHCDI-Net' that integrates Convolutional Neural Networks (CNNs) and Transformers for low-light image enhancement (LLIE). The paper is well written.

Replies: We sincerely appreciate Reviewer for your comments and recognition of our manuscript. We are delighted to learn that our paper is considered well-written, which encourages us greatly. We have carefully considered all the suggestions from the reviewers to further improve the quality of our work, and we hope the revised manuscript meets the publication standards.

Attachment Submitted filename: Response Letter.pdf

# Peer review: Decision Letter 1

**DOI:** 10.1371/journal.pone.0352326.r003

**Authors:** Nagaraju Y, Nagaraju Y

17 May 2026

PONE-D-25-60023R1

Enhancing Low-Light Images with MSHCDI-Net: A Multi-Scale Hybrid Cross-Domain Interaction Approach

PLOS One

Dear Dr. Zheng,

Thank you for submitting your manuscript to PLOS ONE. After careful consideration, we feel that it has merit but does not fully meet PLOS ONE’s publication criteria as it currently stands. Therefore, we invite you to submit a revised version of the manuscript that addresses the points raised during the review process.

The reviewers have provided several comments and suggestions regarding the manuscript. In particular, clarification is required for the comparisons presented in Table 1, including the selection of existing models used for evaluation. Additionally, the claims regarding the superiority and effectiveness of the proposed model require further justification.

The authors are kindly requested to carefully address all the reviewers’ comments and revise the manuscript accordingly.

Please submit your revised manuscript by Jul 01 2026 11:59PM. If you will need more time than this to complete your revisions, please reply to this message or contact the journal office at plosone@plos.org. When you're ready to submit your revision, log on to https://www.editorialmanager.com/pone/ and select the 'Submissions Needing Revision' folder to locate your manuscript file.

Please include the following items when submitting your revised manuscript:

- A letter that responds to each point raised by the academic editor and reviewer(s). You should upload this letter as a separate file labeled 'Response to Reviewers'.
- A marked-up copy of your manuscript that highlights changes made to the original version. You should upload this as a separate file labeled 'Revised Manuscript with Track Changes'.
- An unmarked version of your revised paper without tracked changes. You should upload this as a separate file labeled 'Manuscript'.

If you would like to make changes to your financial disclosure, please include your updated statement in your cover letter. Guidelines for resubmitting your figure files are available below the reviewer comments at the end of this letter.

If applicable, we recommend that you deposit your laboratory protocols in protocols.io to enhance the reproducibility of your results. Protocols.io assigns your protocol its own identifier (DOI) so that it can be cited independently in the future. For instructions see: https://journals.plos.org/plosone/s/submission-guidelines#loc-laboratory-protocols. Additionally, PLOS ONE offers an option for publishing peer-reviewed Lab Protocol articles, which describe protocols hosted on protocols.io. Read more information on sharing protocols at https://plos.org/protocols?utm_medium=editorial-email&utm_source=authorletters&utm_campaign=protocols.

As the corresponding author, your ORCID iD is verified in the submission system and will appear in the published article. PLOS supports the use of ORCID, and we encourage all coauthors to register for an ORCID iD and use it as well. Please encourage your coauthors to verify their ORCID iD within the submission system before final acceptance, as unverified ORCID iDs will not appear in the published article. *Only*  the individual author can complete the verification step; PLOS staff *cannot*  verify ORCID iDs on behalf of authors.

We look forward to receiving your revised manuscript.

Kind regards,

Nagaraju Y, Ph.D.

Guest Editor

PLOS One

Journal Requirements:

If the reviewer comments include a recommendation to cite specific previously published works, please review and evaluate these publications to determine whether they are relevant and should be cited. There is no requirement to cite these works unless the editor has indicated otherwise.

Please review your reference list to ensure that it is complete and correct. If you have cited papers that have been retracted, please include the rationale for doing so in the manuscript text, or remove these references and replace them with relevant current references. Any changes to the reference list should be mentioned in the rebuttal letter that accompanies your revised manuscript. If you need to cite a retracted article, indicate the article’s retracted status in the References list and also include a citation and full reference for the retraction notice.

[Note: HTML markup is below. Please do not edit.]

Reviewers' comments:

Reviewer's Responses to Questions

**Comments to the Author**

1. If the authors have adequately addressed your comments raised in a previous round of review and you feel that this manuscript is now acceptable for publication, you may indicate that here to bypass the “Comments to the Author” section, enter your conflict of interest statement in the “Confidential to Editor” section, and submit your "Accept" recommendation.

Reviewer #4: All comments have been addressed

Reviewer #5: (No Response)

**********

2. Is the manuscript technically sound, and do the data support the conclusions?

The manuscript must describe a technically sound piece of scientific research with data that supports the conclusions. Experiments must have been conducted rigorously, with appropriate controls, replication, and sample sizes. The conclusions must be drawn appropriately based on the data presented.

Reviewer #4: Yes

Reviewer #5: Partly

**********

3. Has the statistical analysis been performed appropriately and rigorously?

Reviewer #4: Yes

Reviewer #5: Yes

**********

4. Have the authors made all data underlying the findings in their manuscript fully available?

The PLOS Data policy requires authors to make all data underlying the findings described in their manuscript fully available without restriction, with rare exception (please refer to the Data Availability Statement in the manuscript PDF file). The data should be provided as part of the manuscript or its supporting information, or deposited to a public repository. For example, in addition to summary statistics, the data points behind means, medians and variance measures should be available. If there are restrictions on publicly sharing data—e.g. participant privacy or use of data from a third party—those must be specified.

Reviewer #4: (No Response)

Reviewer #5: (No Response)

**********

5. Is the manuscript presented in an intelligible fashion and written in standard English?

PLOS ONE does not copyedit accepted manuscripts, so the language in submitted articles must be clear, correct, and unambiguous. Any typographical or grammatical errors should be corrected at revision, so please note any specific errors here.

Reviewer #4: Yes

Reviewer #5: Yes

**********

6. Review Comments to the Author

Please use the space provided to explain your answers to the questions above. You may also include additional comments for the author, including concerns about dual publication, research ethics, or publication ethics. (Please upload your review as an attachment if it exceeds 20,000 characters)

Reviewer #4: I appreciate the authors’ effort in preparing the manuscript; however, the incorporation of the following comments is recommended before its final publication.

1. The manuscript contains minor issues related to justification and typographical errors that should be addressed.

2. In Table 1, the comparison between the proposed model and existing models requires further clarification. Some of the referenced models are from conference publications, while others are not recent. The authors are requested to justify the selection of these models for comparison.

3. Additionally, the proposed model does not demonstrate superior performance across all aspects. The authors are therefore requested to provide adequate justification for these claims, particularly with reference to Table 1.

Reviewer #5: The paper is very interesting. But, i have some concerns to express:

1) In Section 5, under 5.1 subsection there is a heading of Dataset. But, the description of it is missing.

2) In Some places, there is write-up error (e.g: under 4.6 subsection, the "does not" is used twice). Please check

3) Keep Consistent wording in entire paper (e.g: KinD or KIND).

4) Please check the alignments of words, space and other grammatical.

**********

7. PLOS authors have the option to publish the peer review history of their article (what does this mean?). If published, this will include your full peer review and any attached files.

If you choose “no”, your identity will remain anonymous but your review may still be made public.

**Do you want your identity to be public for this peer review?**  For information about this choice, including consent withdrawal, please see our Privacy Policy.

Reviewer #4: No

Reviewer #5: **Yes:** Dr. Neetha P U

**********

[NOTE: If reviewer comments were submitted as an attachment file, they will be attached to this email and accessible via the submission site. Please log into your account, locate the manuscript record, and check for the action link "View Attachments". If this link does not appear, there are no attachment files.]

To ensure your figures meet our technical requirements, please review our figure guidelines: https://journals.plos.org/plosone/s/figures

You may also use PLOS’s free figure tool, NAAS, to help you prepare publication quality figures: https://journals.plos.org/plosone/s/figures#loc-tools-for-figure-preparation.

NAAS will assess whether your figures meet our technical requirements by comparing each figure against our figure specifications.

Attachment Submitted filename: PLOP.docx

# Author comment: Author response to Decision Letter 2

**DOI:** 10.1371/journal.pone.0352326.r004

21 May 2026

Replies to Reviewer 4’s Comments

Comments:

1. The manuscript contains minor issues related to justification and typographical errors that should be addressed.

Replies: We sincerely apologize for making such mistakes. We have carefully gone through the entire manuscript again and corrected the typographical errors and formatting issues accordingly.

2. In Table 1, the comparison between the proposed model and existing models requires further clarification. Some of the referenced models are from conference publications, while others are not recent. The authors are requested to justify the selection of these models for comparison.

Replies: We sincerely thank the reviewer for this valuable comment. In the revised manuscript, we have further clarified the rationale for selecting the comparison methods in Section 5.2.These methods cover the main LLIE paradigms. Specifically, Retinex-Net, KinD, MIRNet, and SNR-Net are CNN-based approaches; EnlightenGAN is a GAN-based method; LLFormer is a Transformer-based model; and SNR-Net and HVI-CIDNet further represent hybrid architectures combining CNN and Transformer components.This selection enables a comprehensive evaluation across different design philosophies, facilitating a balanced and fair comparison with state-of-the-art LLIE methods.

3.Additionally, the proposed model does not demonstrate superior performance across all aspects. The authors are therefore requested to provide adequate justification for these claims, particularly with reference to Table 1.

Replies: We sincerely thank the reviewer for this insightful comment. We agree that the proposed method does not achieve the best performance on every individual metric. In the revised manuscript, we have clarified this point and provided a more balanced discussion with respect to Table 1. Specifically, the proposed model is designed to achieve an overall trade-off between enhancement quality and visual naturalness, rather than optimizing a single metric in isolation.

Replies to Reviewer 5’s Comments

Comments:

1.In Section 5, under 5.1 subsection there is a heading of Dataset. But, the description of it is missing.

Replies: Thank you for pointing this out. We have added a dataset description in Section 5.1, introducing LOL-v1, LOL-v2-real, and LOL-v2-synthetic and explaining their roles in evaluating the performance, robustness, and generalization ability of the proposed method.

2.In some places, there is write-up error, e.g., under 4.6 subsection, the “does not” is used twice. Please check.

Rplies: Very sorry for making such mistakes, we have carefully reviewed the entire manuscript and corrected identified issues.

3. Keep consistent wording in entire paper, e.g., KinD or KIND.

Replies: Thank you for the suggestion. We have checked the entire manuscript and unified the naming of this method as “KinD” throughout the paper.

4. Please check the alignments of words, space and other grammatical.

Replies: Thank you for this comment. We have carefully checked the manuscript and corrected spacing, punctuation, alignment, and grammatical issues.

Attachment Submitted filename: Response Letter_20260521162457.pdf

# Editor report: Decision Letter 2

**DOI:** 10.1371/journal.pone.0352326.r005

**Authors:** Nagaraju Y, Nagaraju Y, Nagaraju Y

10 Jun 2026

Enhancing Low-Light Images with MSHCDI-Net: A Multi-Scale Hybrid Cross-Domain Interaction Approach

PONE-D-25-60023R2

Dear Dr. Zheng,

We’re pleased to inform you that your manuscript has been judged scientifically suitable for publication and will be formally accepted for publication once it meets all outstanding technical requirements.

Within one week, you’ll receive an e-mail detailing the required amendments. When these have been addressed, you’ll receive a formal acceptance letter and your manuscript will be scheduled for publication.

An invoice will be generated when your article is formally accepted. Please note, if your institution has a publishing partnership with PLOS and your article meets the relevant criteria, all or part of your publication costs will be covered. Please make sure your user information is up-to-date by logging into Editorial Manager at Editorial Manager® and clicking the ‘Update My Information' link at the top of the page. For questions related to billing, please contact billing support.

If your institution or institutions have a press office, please notify them about your upcoming paper to help maximize its impact. If they’ll be preparing press materials, please inform our press team as soon as possible -- no later than 48 hours after receiving the formal acceptance. Your manuscript will remain under strict press embargo until 2 pm Eastern Time on the date of publication. For more information, please contact onepress@plos.org.

Kind regards,

Nagaraju Y, Ph.D.

Guest Editor

PLOS One

Additional Editor Comments (optional):

Reviewers' comments:

All reviewer comments have been adequately addressed by the authors. The revised manuscript has been substantially improved, and no further concerns remain. I recommend the manuscript for publication in its present form.

# Editor report: Acceptance letter

**DOI:** 10.1371/journal.pone.0352326.r006

**Authors:** Nagaraju Y, Nagaraju Y, Nagaraju Y

PONE-D-25-60023R2

PLOS One

Dear Dr. Zheng,

I'm pleased to inform you that your manuscript has been deemed suitable for publication in PLOS One. Congratulations! Your manuscript is now being handed over to our production team.

At this stage, our production department will prepare your paper for publication. This includes ensuring the following:

* All references, tables, and figures are properly cited

* All relevant supporting information is included in the manuscript submission,

* There are no issues that prevent the paper from being properly typeset

You will receive further instructions from the production team, including instructions on how to review your proof when it is ready. Please keep in mind that we are working through a large volume of accepted articles, so please give us a few days to review your paper and let you know the next and final steps.

Lastly, if your institution or institutions have a press office, please let them know about your upcoming paper now to help maximize its impact. If they'll be preparing press materials, please inform our press team within the next 48 hours. Your manuscript will remain under strict press embargo until 2 pm Eastern Time on the date of publication. For more information, please contact onepress@plos.org.

You will receive an invoice from PLOS for your publication fee after your manuscript has reached the completed accept phase. If you receive an email requesting payment before acceptance or for any other service, this may be a phishing scheme. Learn how to identify phishing emails and protect your accounts at https://explore.plos.org/phishing.

If we can help with anything else, please email us at customercare@plos.org.

Thank you for submitting your work to PLOS ONE and supporting open access.

Kind regards,

PLOS ONE Editorial Office Staff

on behalf of

Dr. Nagaraju Y

Guest Editor

PLOS One
